# Electrical Current Map and Bulk Conductivity of Carbon Fiber-Reinforced Nanocomposites

**DOI:** 10.3390/polym11111865

**Published:** 2019-11-12

**Authors:** Liberata Guadagno, Luigi Vertuccio, Carlo Naddeo, Marialuigia Raimondo, Giuseppina Barra, Felice De Nicola, Ruggero Volponi, Patrizia Lamberti, Giovanni Spinelli, Vincenzo Tucci

**Affiliations:** 1 Department of Industrial Engineering, University of Salerno, Via Giovanni Paolo II, 132, 84084 Fisciano (SA), Italy; lvertuccio@unisa.it (L.V.); cnaddeo@unisa.it (C.N.); gbarra@unisa.it (G.B.); 2 CIRA Italian Aerospace Research Centre, Advanced Materials and Technologies Lab, 81043 Capua (CE), Italy; fdenicola@cira.it (F.D.N.); rvolponi@cira.it (R.V.); 3 Department of Information and Electrical Engineering and Applied Mathematics, University of Salerno, Via Giovanni Paolo II, 132, 84084 Fisciano (SA), Italy; plamberti@unisa.it (P.L.); gspinelli@unisa.it (G.S.); vtucci@unisa.it (V.T.)

**Keywords:** carbon–carbon composites (CCCS), particle reinforcement, structural composites, electrical properties, tunneling atomic force microscopy (TUNA) technique, thermosetting resins

## Abstract

A suitably modified resin film infusion (RFI) process was used for manufacturing carbon fiber-reinforced composites (CFRCs) impregnated with a resin containing nanocages of glycidyl polyhedral oligomeric silsesquioxane (GPOSS) for enhancing flame resistance and multi-wall carbon nanotubes (MWCNTs) to contrast the electrical insulating properties of the epoxy resin. The effects of the different numbers (7, 14 and 24) of the plies on the equivalent direct current (DC) and alternating current (AC) electrical conductivity were evaluated. All the manufactured panels manifest very high values in electrical conductivity. Besides, for the first time, CFRC strings were analyzed by tunneling atomic force microscopy (TUNA) technique. The electrical current maps highlight electrically conductive three-dimensional networks incorporated in the resin through the plies of the panels. The highest equivalent bulk conductivity is shown by the seven-ply panel characterized by the parallel (σ_//0°_) in-plane conductivity of 16.19 kS/m. Electrical tests also evidence that the presence of GPOSS preserves the AC electrical stability of the panels.

## 1. Introduction

To fully apply composite materials in wide extent in aircraft vehicles, several drawbacks still need to be overcome, such as a suitable electrical conductivity to better cover several functions (e.g., lightning strike and electromagnetic compatibility issues) and continuous enhancement of fire safety in aviation materials. Many relevant achievements have been described in the literature in this direction [1,2,3,4,5,6,7,8,9,10]. Many functions can be conferred to aeronautical composites by proper use of nanotechnology, for example by embedding nanostructured materials with exceptional properties, like carbon nanotubes (CNTs), in aeronautical grade epoxy resins before or during the manufacturing processes [11,12,13,14,15,16]. The introduction of secondary nanoscale reinforcements (e.g., graphene, carbon nanotubes or nanoclay distributed in polymer matrix or fiber sizing) into the fiber-reinforced composites may contribute to further improvements. In a recent paper, Zhou et al. showed that the addition of secondary CNT nano reinforcement into epoxy matrices ensures a strong increase of the composite strength [17]. In the same way, the addition of carbon nanotubes has an effect on the electrical properties of epoxy resins that can be suitably enhanced to reach the typical values (around 10 S/m) required for the structural parts (fuselage, wings, etc.) of modern aircrafts. The main technology adopted to manufacture such parts relies on the use of multiple plies of suitably impregnated carbon fiber-reinforced (CFR) preform. Many papers deal with the behavior of resin formulations adopted for the impregnation of CFRCs [18,19,20,21,22,23]. In order to improve the electrical conductivity of insulating resins, as epoxies, typically adopted in the manufacturing processes for aeronautical applications, a small weight percentage of electrically conducting fillers can be added [2,19,20,24,25,26]. The notable performance of CNTs, which are also capable of enhancing the desired photo-oxidative stability of the final material [27], makes this nanostructured form of carbon very appealing to achieve such a goal. The conductive behavior of the CNT-epoxy mixture is due to the formation of a percolation network of conducting nanoparticles even at a low filler amount [28,29,30,31]. The low CNT percentages necessary to achieve good electrical properties of the impregnating resin have led to relevant interest for the realization of CFRCs. In particular, for CNT-based CFRCs, a fundamental aspect is the interplay between the processing approach and the electrical response of CNT-based nanocomposite systems [19,20,32]. Due to the requirements imposed by the application context, issues related to the flame resistance properties have to be also addressed. Many authors have studied flame-retardant epoxy resins [1,2,3,33,34,35,36]. Aeronautical epoxy resins and their hardener agents containing phosphorus have been synthesized and used to prepare epoxy formulations and CFRCs [1,3,37,38]. Recently, CFRCs have been manufactured adopting a modified resin film infusion (RFI) process (named “bulk infusion”) [20,39,40]. In particular, the difference in the electrical performance of carbon fiber-reinforced composites (CFRCs) made with two different resin film infusion (RFI) manufacturing techniques has been analyzed. For the panel obtained by bulk infusion, the equivalent conductivity measured along the carbon fibers (in-plane) and that derived in direction perpendicular to the fibers (out-of-plane) were 2.0 × 10^4^ and 3.9 S/m, respectively, whereas by using the traditional resin film infusion the in-plane and out-of-plane values were 1.1 × 10^4^ and 1.7 S/m, respectively. Morphological investigations highlighted that this difference in the electrical conductivity is strictly related to the different distribution of multiwall carbon nanotubes (MWCNTs) between the carbon fibers (CFs) of the plies [19]. The main aim of this paper is to formulate a composite material, suitable for advanced structural applications, able to overcome the lack of a suitable electrical conductivity to better cover several functions such as antistatic properties, lightning strike protection and electromagnetic compatibility issues. For this purpose, a formulation containing GPOSS nanocages (which has already demonstrated enhanced flame resistance) has been used [1,2]. In particular, in this paper, electrical measurements were carried out on several flat panels characterized by different thicknesses and number of plies, manufactured with resin loaded with MWCNTs, and containing GPOSS as the flame-resistant agent. The values of both in-plane and out-of-plane equivalent electrical conductivities were analyzed together with the electrical current map of the panels. The electrical response to the applied voltage provides satisfying results. The curing degree of the resin is suitable to meet industrial requirements.

## 2. Experimental 

### 2.1. Materials

A mixture of tetraglycidyl methylene dianiline (TGMDA) and 1,4-butanedioldiglycidylether (BDE) in the ratio 75:25 wt % was used as epoxy precursor. Both components were obtained from Sigma-Aldrich (Milan, Italy). NANOCYL® NC3100™ series multiwall carbon nanotubes, produced via the catalytic chemical vapor deposition (CCVD) process, with purity higher than 95%, were purchased from Nanocyl (Sambreville, Belgium); glycidyl oligomeric silsesquioxanes (GPOSS) was bought from Hybrid Plastic (Hattiesburg, MS, USA). 4,4′-diaminodiphenyl sulfone (DDS), purchased from Sigma-Aldrich (Milan, Italy), was used as the hardener agent. Plain weave carbon cloth fabric (HEXCEL HexForce ® G0814 6 1000 TCT - HEXCEL, Saronno (VA), Italy) with a thickness of 0.2 mm and with an areal density of 0.193 kg/m^2^ was used for the preparation of CFRCs.

#### Epoxy Formulations D and E (Without CFs)

Epoxy blend (TGMDA and BDE) was mixed at 90 °C. At this temperature, carbon nanotubes, (0.5% by weight) and/or POSS compounds (5% by weight) were added and dispersed using a Hielscher model UP200S (200 W, 24 kHz) (Hielscher Ultrasonics GmbH, Teltow, Germany) ultrasound. After 20 min, the temperature was raised to 120 °C and the hardener DDS was added. The epoxy system was mixed at this temperature until the complete solubilization of the hardener. For producing the coupons for the tests, the epoxy mixture was cooled down, poured in molds of opportune dimensions and solidified using a two-stage curing cycle: the first at the lower temperature of 125 °C for 1 h and the second one at 200 °C for 3 h. In this paper, the formulation containing only CNTs was named sample D, and the formulation containing both CNTs and GPOSS was named sample E. Formulation E (0.5% by weight of MWCNTs and 5% by weight of GPOSS) was used for the manufacturing of the CFRCs.

The concentration of 0.5 wt % of MWCNTs was selected because, for this concentration, the sample is beyond the electrical percolation threshold (EPT). The presence of 0.5 wt % of MWCNTs and 5.0 wt % of GPOSS causes an enhancement in the DC volume conductivity from 8.00 × 10^−13^ (unfilled formulation) to 3.54 × 10^−3^ S m^−1^. The measurements of the DC conductivity were performed on disk-shaped specimens of about 2 mm thickness and 50 mm diameter by using circular metalized electrodes with a diameter of about 22 mm following an experimental procedure already described in the literature [24]. 

A blank formulation (pristine) without carbon nanotubes and GPOSS was also prepared. Table 1 shows the prepared formulations.

### 2.2. Carbon Fiber-Reinforced Composites (CFRCs)

**Figure 1:**
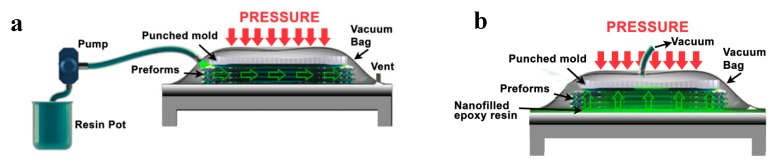
Scheme of the (**a**) classic and (**b**) modified liquid resin infusion techniques.

CFRCs were manufactured by a non-usual resin film infusion (RFI) process, described in previous papers [20,39]. A laminate configuration (0/90)*_n_* was used for the purpose. In the classic RFI, a dry carbon fiber preform is placed in a vacuum bag and the fluid nanofilled resin is injected from an edge of preform, while in the other the vacuum is vent so the resin flows through the length of the preform as in the scheme of Figure 1a. 

The classical infusion process requires very low viscosities of the resin. Usually, the value of viscosity for this process is lower than 0.3 Pa s. In literature, it is also possible to find works performed with resins reaching the theoretical maximum limit of viscosities around 0.8 Pa s. For suitable values of viscosities, processes based on RFI are versatile and potentially cost-effective but, when the resin contains nanoparticles, the severe increase of the viscosity and the filtering effects cause its injection in a dense fiber reinforcement to be very difficult, making it impossible to obtain homogeneous final products of good quality. For this reason, a modified RFI was performed in this paper with the aim of overcoming the severe process limit conditions, through an experimental trade-off assessment of properties and parameters. With this procedure, the resin was forced to flow through the thickness of the preform using an external vacuum pump (see Figure 1b). Very promising results have been obtained by the combination of thick wet film and infusion under vacuum bag in autoclave. This procedure has allowed obtaining CFRCs of adequate quality, with a satisfying resin content (%), low void content, a low filtering effect and limited costs.

#### CFRCs—Manufacturing Process (Panels D, E1, E2 and E3)

The thick film of nanocharged resin was placed over a release film (Release Ease 234 TFP-HP Airtech). Then, a dry preform (400 mm × 400 mm) made by laminating seven plies of carbon fiber cloths was placed on the resin thick film (see Figure S1a in Supplementary Materials). The edges of the preform were sealed to force the resin to flow through the thickness. The laminate was covered by a porous release film and a distribution medium to allow the resin to escape from the upper side. A bleeder medium was placed around the preform to receive the excess of resin (see Figure S1b,c in Supplementary Materials). Finally, a vacuum bag was prepared. The closed laminate was placed in the autoclave (see Figure S1d in Supplementary Materials). The curing cycle has been set as follows: (1) the first ramp to 120 °C at 2 °C/min, vacuum active; (2) dwell of 30 min at 120 °C; (3) pressure at 7 bar, vent open; (4) a second ramp to 180 °C at 2 °C/min and (5) dwell of three hours at 180 °C. The squeezing of resin, after the curing process, is shown in Figure S1e in Supplementary Materials. The bleeder/breather absorbed the excess of resin. The obtained panel, after the curing process, is shown in Figure S1f in Supplementary Materials. Two panels with seven plies (E1) were prepared using this procedure. In particular, two panels with thicknesses of 1.39 and 1.35 mm, respectively, with a calculated volume fiber fraction of *V*_f_ = 0.55, were manufactured. The same procedure was used to produce panels with 14 plies (E2) and 24 plies (E3). The panels were cut to obtain the samples on which electrical measurements were carried out. Two types of samples were considered, namely (rectangular) strips and squares, as described in Section 2.3.6. Sample D, without the flame-retardant compound (GPOSS), was also manufactured using the same manufacturing process employed for E1, E2 and E3 samples with the aim of studying the effect of the flame-retardant component on the electrical conductivity. Table 2 summarizes characteristics and composition of the materials manufactured by using the modified RFI analyzed in this work.

### 2.3. Methods

#### 2.3.1. Differential Scanning Calorimetry (DSC) Thermal Analysis

The curing degree of the epoxy formulation E used for manufacturing E1, E2 and E3 panels was evaluated by differential scanning calorimetry. Mettler DSC 822/400 (Mettler-Toledo, Columbus, OH, USA) thermal analyzer was used to determine the heat of the reaction on both cured and uncured samples on the assumption that the exothermic heat (Δ*H)* during the dynamic measurement is proportional to the extent of the curing reaction [41]. The curing degree is given from the following equation:

(1) $$DC=\;\frac{\Delta H_{T}-\Delta H_{\mathrm{Res}}}{\Delta H_{T}}\;\times 100$$

where Δ*H*_T_ is the total heat of reaction of the uncured material and Δ*H*_Res_ is the residual heat of reaction of the panels cured in the autoclave.

#### 2.3.2. Rheological Measurements

Rheological measurements in oscillation mode were performed using a Rheometer AR 2000 (TA Instruments, Zürich, Switzerland). Parallel plates with a diameter of 40 mm were selected as an appropriate geometry and the gap was set at a value of 300 mm. Temperature sweeps from 50 to 120 °C were carried out at constant frequency of 1 Hz at a strain percent set at 5% within the linear viscoelastic region (determined by strain sweep test). The results were shown as curves of the complex viscosity η* as a function of the temperature.

#### 2.3.3. Morphological Analysis: Field Emission Scanning Electron Microscopy (FESEM) and High-Resolution Transmission Electron Microscopy (HRTEM)

The morphologies of the carbon nanotubes (MWCNTs) and nanocomposite corresponding to the formulation E as well as those of the CFRCs based on the formulation E (panels E1, E2 and F3) were investigated after etching procedure to remove a fraction of the resin around the nanofiller and therefore to better evidence the morphological features [19,20]. Micrographs were obtained by using a field emission scanning electron microscope (FESEM, mod. LEO 1525, Carl Zeiss SMT AG, Oberko-chen, Germany). Strips of CFRCs were cut out from the panels and analyzed in the directions parallel (i.e., in-plane) and perpendicular (i.e., out-of-plane) to the panel plane. High-resolution transmission electron microscopy (HRTEM) characterization of the carbon nanotubes was performed on a Jeol 2010 LaBa_6_ microscope operating at 200 kV. MWCNTs were dispersed (in ethanol) by ultrasonic waves for 30 min. The obtained suspension was dropped on a copper grid (holey carbon).

#### 2.3.4. Energy Dispersive X-ray (EDX) Analysis

The dispersion of polyhedral oligomeric silsesquioxane (GPOSS) nanocages in the nanocomposite corresponding to the formulation E was evaluated using an Energy Dispersive X-ray analyzer (EDX mod. INCA Energy 350, Oxford Instruments, Witney, UK), using the signal of sodium atoms. Before the evaluation of the elemental composition, the samples were coated with chromium (layer thickness 150 Ǻ) using a turbo sputter coater (mod. K575X, EmiTech Ashford, Kent, UK).

#### 2.3.5. Tunneling Atomic Force Microscopy (TUNA) Measurements

TUNA investigation was performed on panels E1, E2 and E3 before and after the etching procedure, whereas sample E was analyzed only after the etching procedure. TUNA is a contact mode technique in which the tip is in uninterrupted contact with the sample. The measurements require the use of an electrically conductive tip of 20 nm and platinum-coated probes with nominal spring constants of 35 Nm^−1^. During the measurements, the DC sample bias was set to values between 1 and 3 V, which are really low values if we consider that the limit for this parameter is 12 V; the current sensitivity was 1pA/V; the scan rate was set to 1.00 Hz s^−1^, halving the value to 0.500 Hz s^−1^ in order to improve the image quality; the number of samples per ramp was 256 reaching up to 512 to get higher resolution images. It is worth noting that, due to the high values of the electrical conductivity of the manufactured panels, the characterization by TUNA was carried out without grounding the samples. Usually, in fact, for this type of measurement carried out on polymeric materials, it is necessary to use the conductive silver paste which ensures adequate electrical contacts of the sample with the ground. The four images of height, deflection error, friction and tuna current, collected simultaneously, were examined using the Bruker software Nanoscope Analysis 1.80 (BuildR1.126200). 

#### 2.3.6. Electrical Measurements

**Figure 2:**
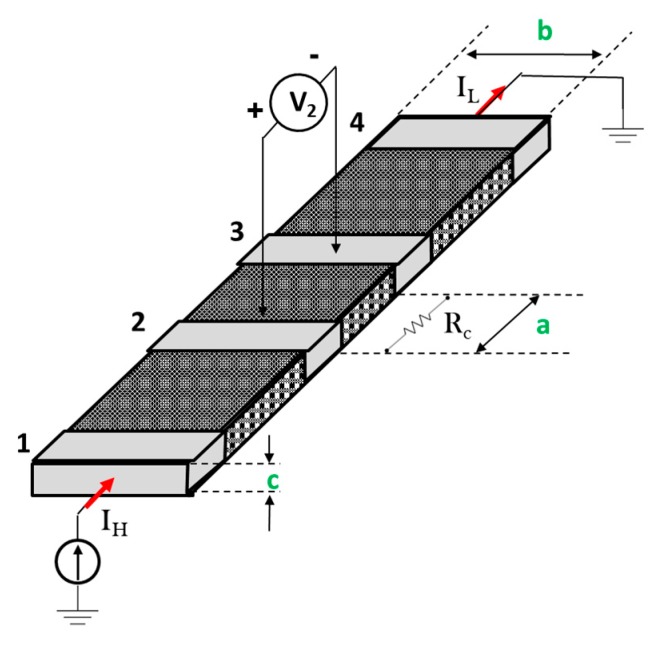
Set-up for the measurement of the in-plane “equivalent” DC conductivity.

DC electrical properties and AC electrical behavior of the panels listed in Table 2 were evaluated. DC electrical conductivity values were obtained using two multimeters (HP 34401A, HP 3408A, Loveland, CO, USA) and an electrometer (Keithley 6514A, Cleveland, OH, USA). The use of the QUADTECK multimeter allowed to investigate the AC electrical behavior in the frequency range 10 Hz to 1 MHz. As concerns the DC characteristics, bulk and surface (σ*_S_*) DC conductivities have been investigated for the resulting CFRCs. In particular, it is possible to extract in-plane and out-of-plane “equivalent” DC bulk-conductivity only after the definition of the sample geometry. The in-plane equivalent conductivity of the CFRCs was obtained by adopting the four-probe method on strip samples of about 4.0 × *b* × *c* cm^3^ with the width *b* = 1.7 cm and the thickness of sample *c*, changing according to the number of plies. In Figure 2 the schematic of the adopted set-up for the measurement of the in-plane DC conductivity is illustrated.

Here the gray regions numbered from 1 to 4 represent the metallized electrical contacts used in the four-probe methods. The distances between contacts 1–2 and 3–4 were about 6 mm, while that between central contacts 2–3 (i.e., *a* in Figure 2) was about 3 mm. In particular, by applying a voltage source between electrodes 1 and 4, a current *I*_H_ = *I*_L_ flows in the section *b* × *c*, which gives rise to a voltage drop *V*_2_ between contacts 2 and 3. *I*_H_ and *V*_2_ are measured and used to derive *R*_c_ = *V*_2_/*I*_H_, which is the resistance of the particular sample from which the in-plane conductivity $\sigma_{//\;}$can be derived as:

(2) $$\sigma_{//\;}=\frac{1}{R_{c}}*\frac{a}{b*c}.$$

In order to consider the measurement′s chain dependencies, the experimental data will be reported with an error Δσ_//_% given by the following equation:

(3) $$\Delta \sigma_{//}\%=\frac{\Delta \sigma_{//}}{\sigma_{//}}*100=\sqrt{{\left(\frac{\Delta R_{c}}{R_{c}}\right)}^{2}+{\left(\frac{\Delta a}{a}\right)}^{2}+{\left(\frac{\Delta b}{b}\right)}^{2}{\left(\frac{\Delta c}{c}\right)}^{2}}*100.$$

**Figure 3:**
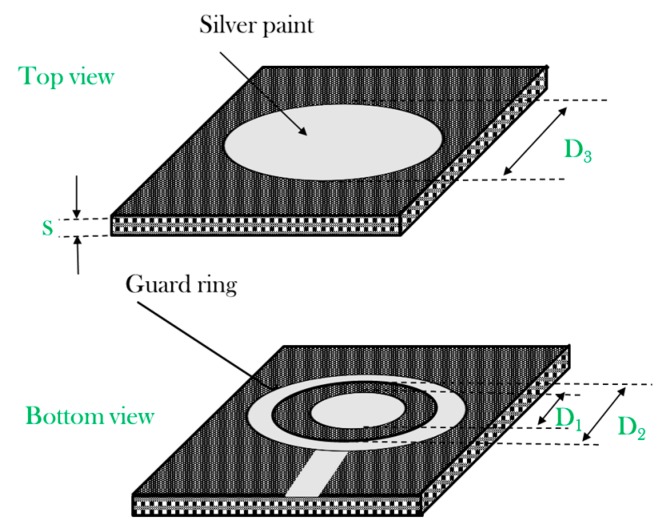
Top and bottom view of the square samples with electrodes (grey area) for three-point measurement technique.

Due to the anisotropy of the material, the resulting conductivity will be a function of the cutting direction. So far, in this paper, the parallel (σ_//0°_) and transverse (σ_//45°_) in-plane conductivity are considered by cutting the sample with the dimension *a* aligned or forming 45° with respect to the fiber′s direction, respectively. The out-of-plane equivalent conductivity of the CFRPs, i.e., σ_﬩_, is evaluated by means of three electrodes (concentric with guard ring configuration) set-up applied to square samples of about 6 × 6 × *s* cm^3^ where the thickness of the sample *s* varies according to the number of plies (Figure 3).

In Figure 3**,** *D*_1_ and *D*_2_ are the diameters of the inner and outer (guard ring) electrodes of the bottom side, respectively, and *D*_3_ is the electrode diameter of the top side. 

**Figure 4:**
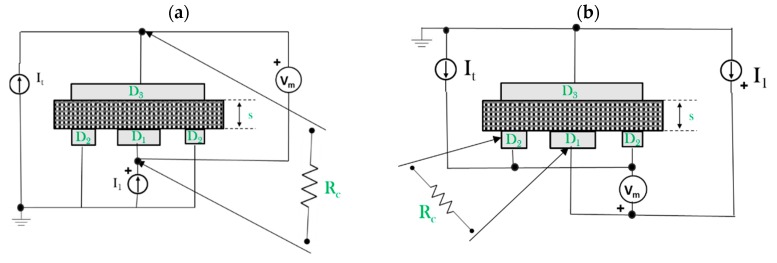
The lateral side of the square sample with set-up measurement of the (**a**) out-of-plane and (**b**) surface conductivity.

By setting *g* and *K*_v_ equal to:

(4) $$g=\frac{D_{2}-D_{1}}{2},$$

(5) $$K_{v}={\left(\frac{D_{1}}{2}+0.5*\frac{g}{2}\right)}^{2},$$

the out-of-plane conductivity $\sigma_{﬩}$ is given by:

(6) $$\sigma_{﬩}=\frac{1}{R_{c}}*\frac{s}{\pi *K_{v}},$$

where *R*_c_ *= V*_m_*/I*_1_ is the ohmic resistance of the sample section of the effective area π × *K*_v_ if the measurement set-up of Figure 4a is used.

The measurement chain will lead to an error $\Delta \sigma_{﬩}\%$ given by:

(7) $$\Delta \sigma_{﬩}\%=\frac{\Delta \sigma_{﬩}}{\sigma_{﬩}}*100=\sqrt{{\left(\frac{\Delta R_{c}}{R_{c}}\right)}^{2}+{\left(\frac{\Delta s}{s}\right)}^{2}+{\left(\frac{\Delta K_{v}}{K_{v}}\right)}^{2}*100},$$

(8) $$\Delta K_{v}=\sqrt{K_{v}}*\sqrt{{\left(\Delta D_{1}\right)}^{2}+{\left(0.5*\Delta g\right)}^{2}},$$

(9) $$\Delta g=\frac{1}{2}*\sqrt{{\left(D_{1}*\Delta D_{2}\right)}^{2}+{\left(D_{2}*\Delta D_{1}\right)}^{2}}.$$

The square sample of Figure 3 was also used to investigate the surface DC conductivity of the CFRPs, i.e., σ_s_. In particular, by adopting the three-probe electrode configuration shown in Figure 4b, the measured resistance *R*_c_ *= V*_m_*/I*_1_ leads to obtain:

(10) $$\sigma_{s}=\frac{1}{R_{c}}*\frac{1}{\pi *K_{s}},$$

where *K*_s_ *= D*_0_*/g* and *D*_0_ *= D*_1_ *+ g*. 

In this case the error Δσ_s_% will be computed according to the following equations:

(11) $$\Delta \sigma_{s}\%=\frac{\Delta \sigma_{s}}{\sigma_{s}}*100=\sqrt{{\left(\frac{\Delta R_{c}}{R_{c}}\right)}^{2}+{\left(\frac{\Delta K_{s}}{K_{s}}\right)}^{2}}*100,$$

(12) $$\Delta K_{s}=K_{s}*\sqrt{{\left(\frac{\Delta D_{0}}{D_{0}}\right)}^{2}+{\left(\frac{\Delta g}{g}\right)}^{2}},$$

(13) $$\Delta D_{0}=\sqrt{{\left(D_{1}*\Delta g\right)}^{2}+{\left(g*\Delta D_{1}\right)}^{2}}.$$

Finally, the AC characteristics were investigated by comparing the impedance of the samples measured according to the setup of Figure 4a. Before performing electrical measurements, the samples were cleaned with acetone and thermally pretreated at 80 °C for 24 h. Then, contacts made with silver paint (Alpha Silver Coated Copper Compound Screening, with a thickness of about 50 µm and a resistivity of 0.7 Ω/sq (square)), according to the particular measurement setup, were deposited on the sample surfaces.

## 3. Results and Discussion

### 3.1. Curing Cycle and Cure Degree of the Resin Impregnating the Panels

**Figure 5:**
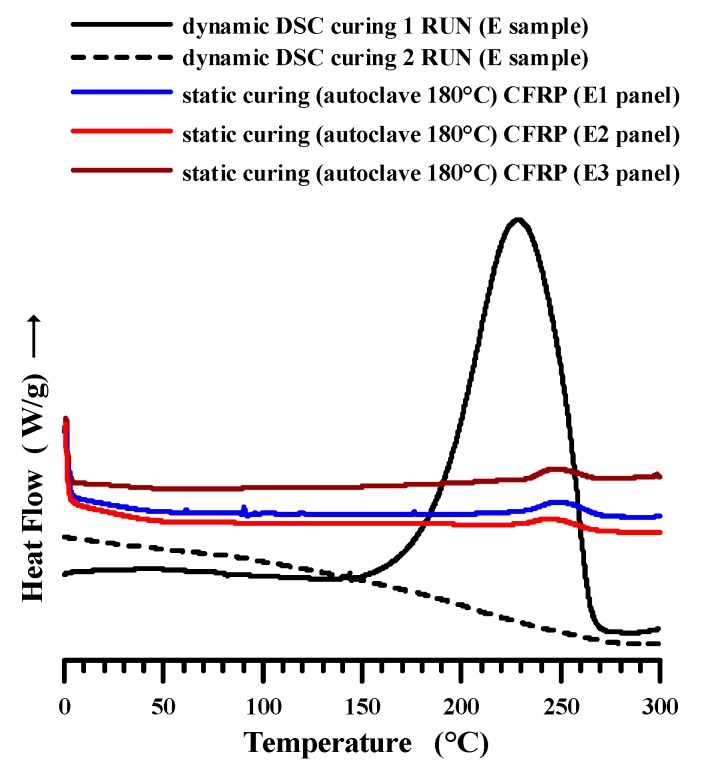
DSC curves of the uncured and cured epoxy formulation E and the panels E1, E2 and E3 after the curing cycle in the autoclave.

The curing degree of the epoxy formulation E used to manufacture the panels E1, E2 and E3 was evaluated using differential scanning calorimetry (DSC). The results of the calorimetric analysis for the formulation E uncured (first run), after dynamic curing cycle (second run) and for E1, E2 and E3 panels are shown in Figure 5.

The determination of the total heat of reaction (Δ*H*_T_) was obtained using a three-step dynamic heating program in the temperature range between −50 and 300 °C.

Sample E (uncured formulation) was firstly scanned at 10 °C/min from 0 to 300 °C (dynamic DSC curing first run, see the black graph in Figure 5), then cooled at 50 °C/min and immediately rescanned from 0 to 300 °C (dynamic DSC curing second run, see the black dotted graph in Figure 5), to verify the eventual presence of residual heat of the reaction. No peaks were observed in the second run, therefore the sample was considered totally cured (DC = 100%) and the exothermic heat (Δ*H)* during the first run was assumed as the total heat of reaction of the uncured material (Δ*H*_T_). 

Panels E1, E2 and E3 were analyzed after the curing in the autoclave by performing the curing cycle up to 180 °C, as described in Section 2.2. The cured panels were scanned at 10 °C/min from 0 to 300 °C (see the colored graphs in Figure 5). The residual heat of reaction Δ*H*_Res_ of the cured panels (E1, E2 and E3) was calculated considering the exotherm peaks observed in the graphs of Figure 5, and the curing degree was evaluated considering Equation 1 described in Section 2.3.1.

The absence of the residual heat of reaction (Δ*H*_resid_) for the formulation E in the second run proves that the sample is completely cured after the first dynamic run. The cure degree for the panels after the curing cycle in the autoclave is shown in Table 3. The resin impregnating the panels manifests high cure degree, between 94% and 96%, which is suitable to satisfy the requirements of the DC of carbon fiber-reinforced resins to be applied for structural (aeronautics, automotive field, etc.) applications. It is very likely that the slightly higher DC for panels containing the highest number of plies is due to a more efficient heat transmission determined by the presence of the carbon fibers in the plies.

### 3.2. Rheological Measurements of Sample E

**Figure 6:**
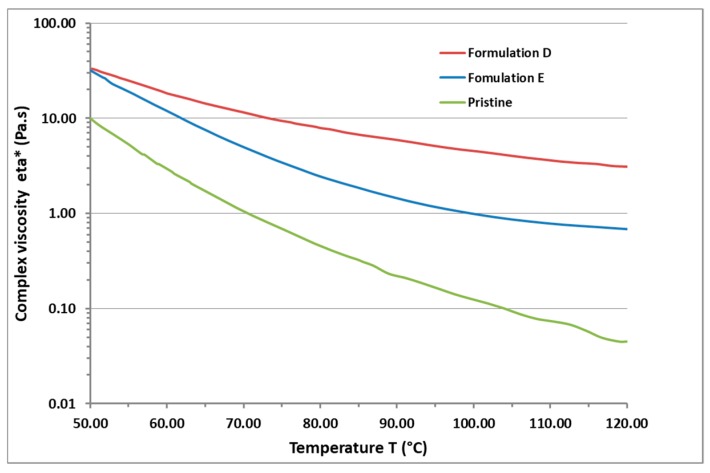
Curves of complex viscosity η* (Pa s) vs. temperature T (°C) for pristine resin, formulation D and formulation E.

Rheological measurements show that GPOSS provides the additional benefit of reducing the viscosity of the epoxy formulations. Figure 6 shows the curve of the complex viscosity η* (Pa s) with the temperature for the pristine resin, the resins filled with 0.5 wt % of MWCNT (formulation D) and the resin filled with 0.5 wt % of MWCNT and 5 wt % GPOSS (formulation E). In all the range of temperature, formulation E, containing GPOSS, is characterized by complex viscosities η* lower than those obtained for the sample containing only CNTs incorporated in the epoxy matrix (formulation D). At the temperature of 120 °C, the complex viscosity of formulation E is lower than 0.7 Pa s, meeting the requirement of viscosity lower than 0.8 Pa s for the infusion process. 

### 3.3. FESEM and HRTEM Morphological Analysis of the Carbon Nanotubes (MWCNTs)

**Figure 7:**
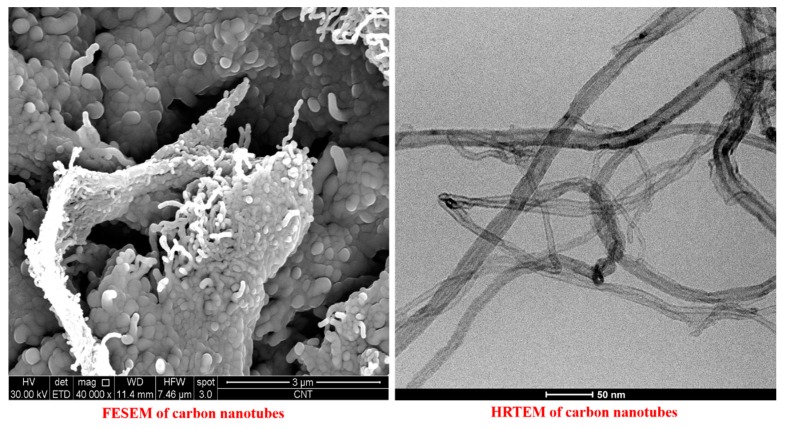
FESEM and HRTEM images of carbon nanotubes (MWCNTs).

FESEM and HRTEM images of the carbon nanotubes are shown in Figure 7. In the FESEM image, MWCNTs appear tightly compacted, forming entangled cords due to strong van der Waals intertube interactions. It is, however, possible to clearly discriminate the carbon nanotubes that emerge in all their length from the interweaving in which they tend to be arranged. The TEM image allows to observe a better separation of the carbon nanotubes, of which it is also possible to easily estimate the diameter. The best separation is due to the dispersion procedure (see Section 2.3.3) that the MWCNTs have undergone, before the HRTEM analysis.

### 3.4. FESEM and TUNA Morphological Analysis of the Nanocomposite TBD + 5% GPOSS + 0.5% MWCNT (Formulation E)

**Figure 8:**
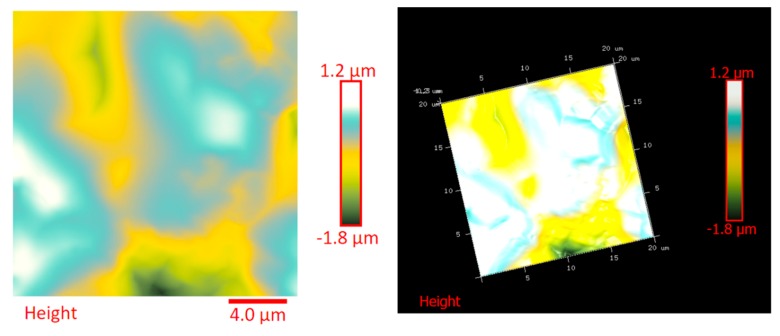
TUNA micrographs (height profiles in 2D and 3D) of the nanocomposite TBD + 5% GPOSS + 0.5% MWCNT (formulation E).

**Figure 9:**
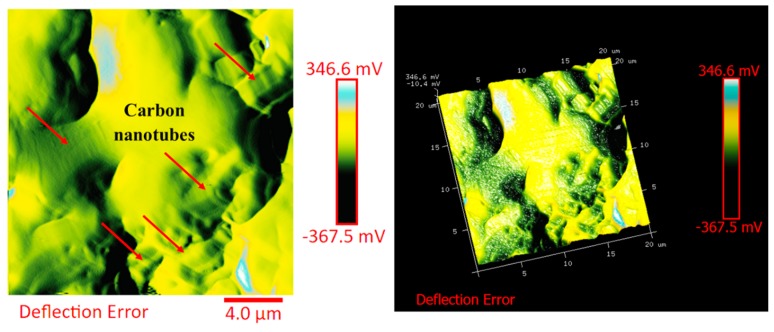
TUNA micrographs (deflection error profiles in 2D and 3D) of the nanocomposite TBD + 5% GPOSS + 0.5% MWCNT (formulation E).

**Figure 10:**
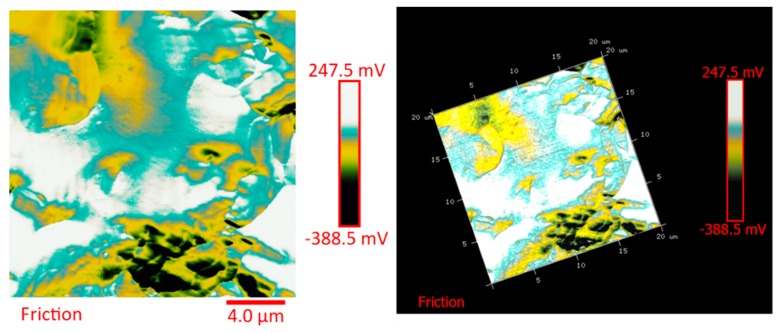
TUNA micrographs (friction profiles in 2D and 3D) of the nanocomposite TBD + 5% GPOSS + 0.5% MWCNT (formulation E).

**Figure 11:**
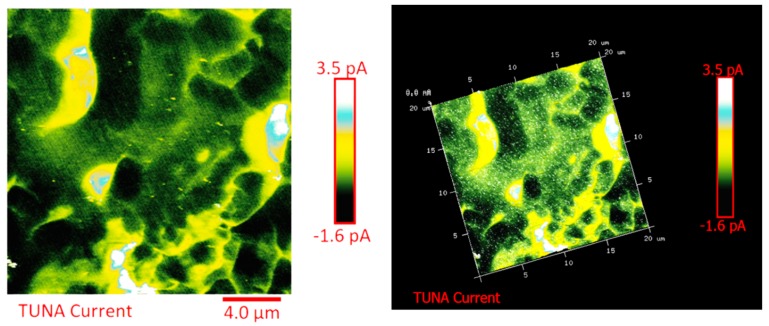
TUNA micrographs (TUNA current profiles in 2D and 3D) of the nanocomposite TBD + 5% GPOSS + 0.5% MWCNT (formulation E).

The distribution of the nanofiller in the epoxy matrix has been first analyzed in the polymeric matrix alone (formulation E) and then in the carbon fiber-reinforced composites (CFRCs) based on the formulation E (panels E1, E2 and E3), after the curing cycle, by means of FESEM and TUNA analysis. Figure S2 in Supplementary Materials shows FESEM images, at different magnifications, corresponding to the formulation E. A dense network of MWCNTs, resembling a stitch frayed in many zones (see higher magnification on the right side) covers all zones of the sample. The carbon nanotubes are able to increase the electrical conductivity of the resin. This has been verified at macroscopic level through measurements of direct electrical conductivity of nanofilled samples [1,19,20]. In this paper, the electrical conductivity of the nanocomposite corresponding to the formulation E has been analyzed at nanoscale level by TUNA investigation. Figure 8, Figure 9, Figure 10 and Figure 11 show, on the left, the TUNA micrographs in 2D (height, deflection error, friction and TUNA current images), and on the right, the corresponding 3D profiles of the epoxy nanofilled sample TBD + 5% GPOSS + 0.5% MWCNT (formulation E). The TUNA technique is very effective in correlating morphological and electrical properties of the carbon-based epoxy resins [2,24] since while the probe is scanning in contact mode, local tunneling current is registered in correspondence of conductive nanodomains, thus giving a homogenous distribution of the carbon nanofiller across the sample surface. MWCNTs appear to firmly cling to the epoxy matrix, as can be seen in all the micrographs shown here, notably in TUNA current images (see Figure 11). Areas of the sample where the presence of conductive carbon nanotubes is more relevant appear brighter than darker areas with lower density of conductive carbon nanotubes. In fact, areas with different luminosities show different current values. In this regard, TUNA current image in Figure 11 allows to discriminate, for the sample TBD + 5% GPOSS + 0.5% MWCNT (formulation E), luminous filaments identifiable with carbon nanotubes and also to detect the presence of low currents ranging from 1.6 to 3.5 pA, confirming the relatively high value of the electrical conductivity (0.168 S/m) of the epoxy nanocomposite and a very good nanofiller dispersion that is revealed by effective conductive paths highlighted by the strong contrast of the colors. In general, however, also the other three types of image, namely height, deflection error and friction, allow to unequivocally distinguish the presence of conductive carbon nanotubes firmly anchored to the polymeric matrix. In particular, the height images show in blue color the “higher (less deep)” areas corresponding to areas with higher density of carbon nanofiller. MWCNTs are also evident in deflection error images; especially in the same areas with higher density of carbon nanotubes (see red arrows). The deflection error image represents the error signal of the deflection parameter that defines the required voltage (and, therefore, the required deflection or force of the cantilever) for the feedback circuit. The deflection error is closely related to the deviation of the vertical deflection from the deflection setpoint that is generated when the tip comes into contact with a particle during the scanning phase of the sample surface. In these conditions, the tip undergoes a slight rebound upwards, causing a slight upward bending of the cantilever with a consequent increase in vertical deflection. However, the feedback circuit can effectively act by returning the vertical deflection to its nominal value using the gain set by the user and sent to the piezo Z to move the tip up or down in order to minimize the error. In the friction images, that allow to detect the frictional forces resulting from the scanning of the AFM probe on the whole sample investigated, the areas with the highest density of carbon nanotubes (effectively stripped by the etching procedure) appear brighter, most probably an index of a higher coefficient of friction than the surrounding substrate, attributable to the conductive filler and its interaction with the host matrix. TUNA images give a map of nanofiller distribution at atomic scale level inside the polymeric matrix, where the carbon nanotubes are integrated into the matrix and become part of the cross-linked structure. 

The CNT/epoxy nanocomposite analyzed shows good electrical properties, which therefore make it appropriate for obtaining CFRCs impregnated with nanofilled resin able to contrast the electrical insulating property of the epoxy resin.

The chemical composition of the nanocomposite TBD + 5% GPOSS + 0.5% MWCNT (formulation E), at the level of microscopic spatial domains, was investigated using the EDX analysis. The EDX images in Figure S3 in Supplementary Materials show the presence of C, O, Si and S, as expected. Their distribution reaches a good level of dispersion; for instance, the image showing the Si element (in yellow color) proves that the GPOSS cages are uniformly distributed in the sample. 

### 3.5. FESEM and TUNA Morphological Analysis of CFRCs (Panels E1, E2 and E3)

**Figure 12:**
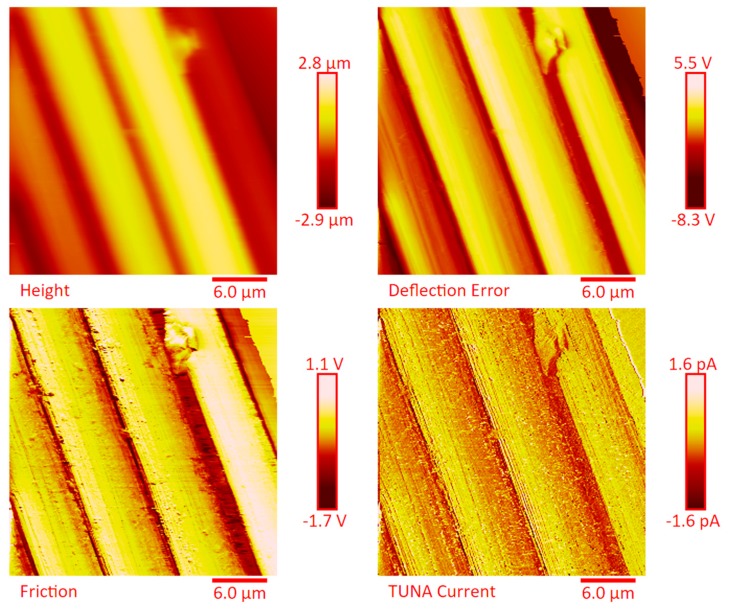
TUNA micrographs (height, deflection error, friction, TUNA current profiles in 2D) of the etched panel E1.

**Figure 13:**
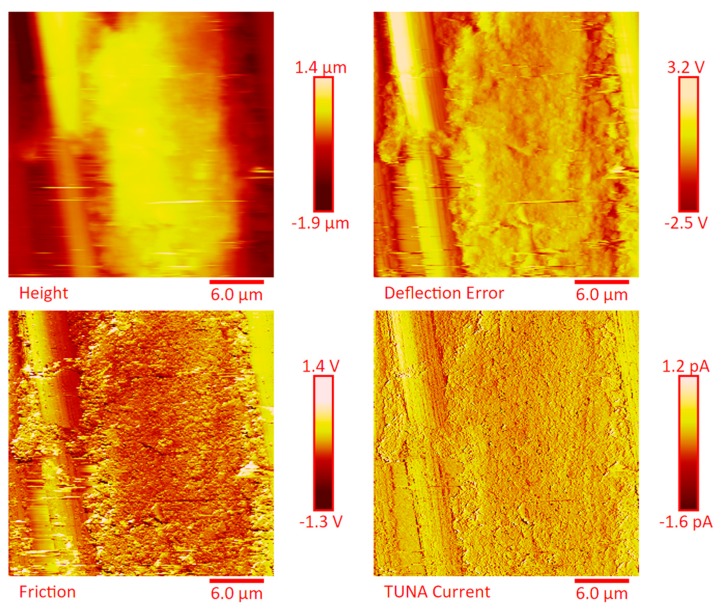
TUNA micrographs (height, deflection error, friction, TUNA current profiles in 2D) of the non-etched panel E1.

**Figure 14:**
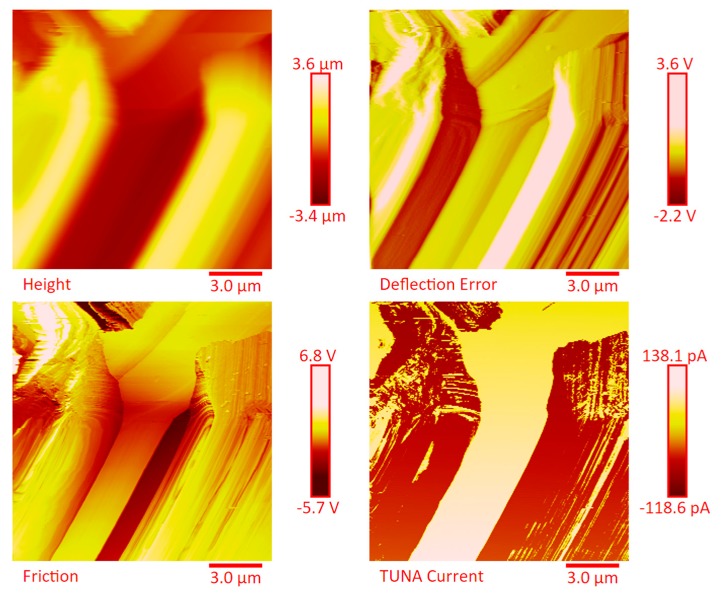
TUNA micrographs (height, deflection error, friction, TUNA current profiles in 2D) of the etched panel E2.

**Figure 15:**
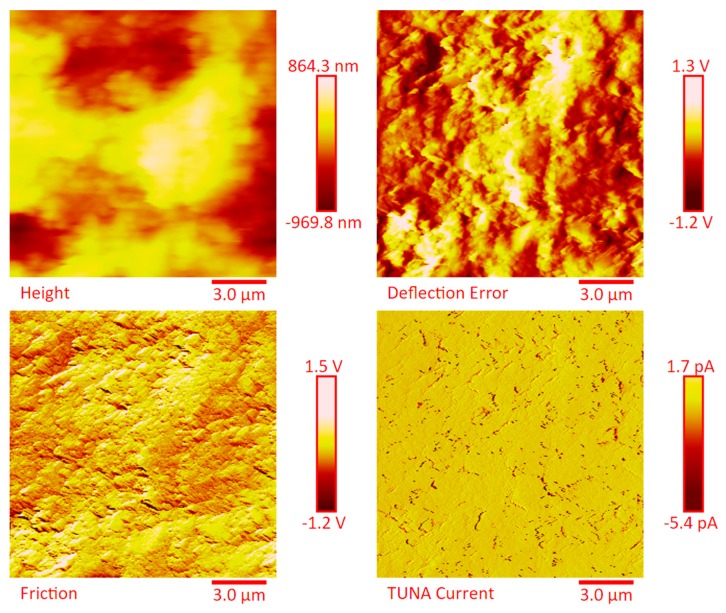
TUNA micrographs (height, deflection error, friction, TUNA current profiles in 2D) of the non-etched panel E2.

**Figure 16:**
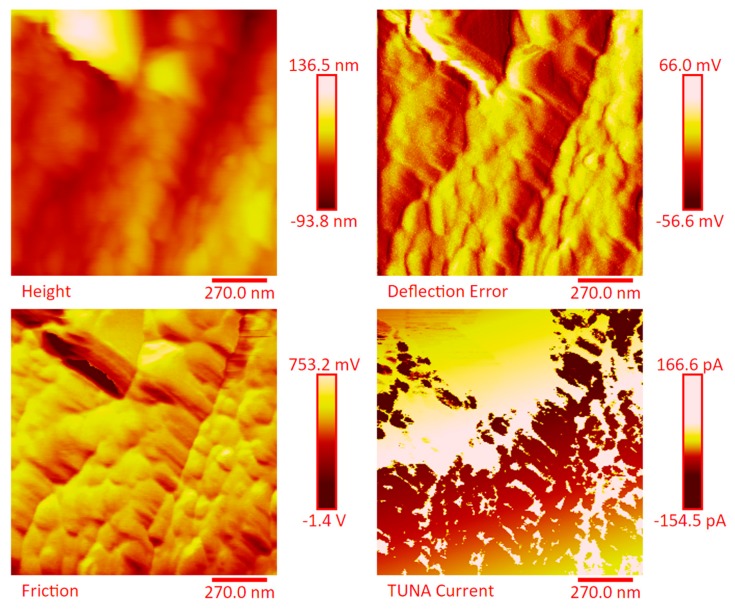
TUNA micrographs at higher magnification (height, deflection error, friction, TUNA current profiles in 2D) of the etched panel E2.

**Figure 17:**
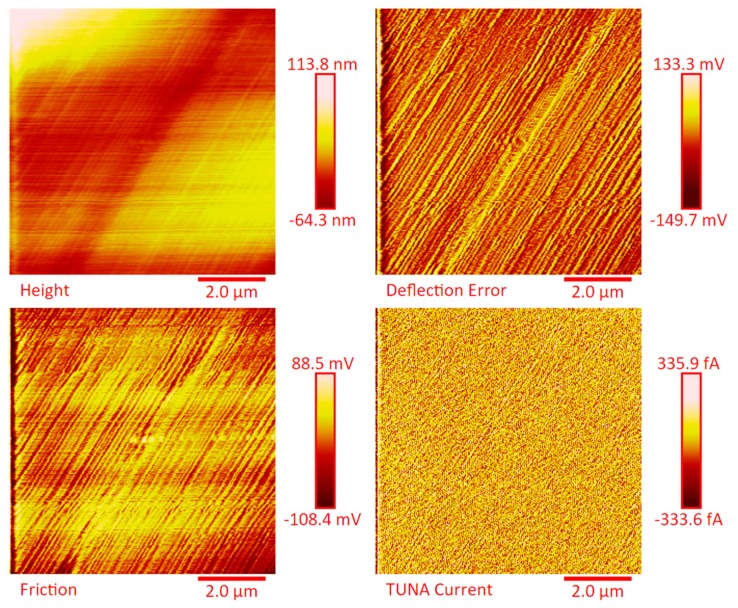
TUNA micrographs (height, deflection error, friction, TUNA current profiles in 2D) of the etched panel E3.

**Figure 18:**
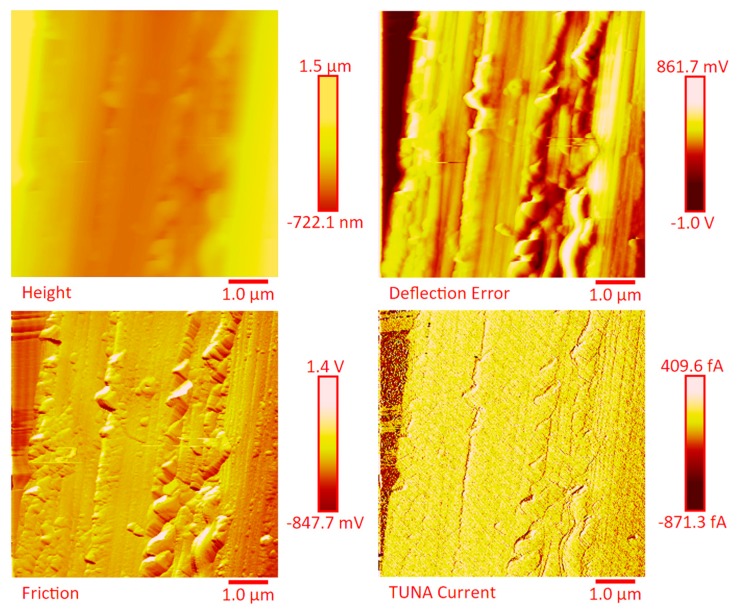
TUNA micrographs (height, deflection error, friction, TUNA current profiles in 2D) of the non-etched panel E3.

The dispersion of the MWCNTs between the plies of the panels was investigated by FESEM after the etching procedure of samples in the form of CFRC strips, which were cut out from the panels and analyzed in the direction perpendicular to the plane of the panels (see Figures S4–S6 in Supplementary Materials). FESEM observations were performed between the plies in the direction perpendicular to the plane to evaluate the performance of the adopted impregnating epoxy system and the efficacy of the implemented infusion process. In particular, the main objective of this investigation was to verify the presence of MWCNTs between the carbon fiber cloths (plies). Choosing the magnifications of Figure S4 corresponding to the magnification 104×, very similar images were obtained for samples E1 and E3 (except for the aspect regarding the number of plies). The images of Figure S4 corresponding to the magnifications 508× and 1018× (on the left side) show different zones at higher magnification of panel E2 (in the direction perpendicular to the panel plane). The images of Figure S4 (on the right side) at higher magnifications show the FESEM images of the E1 panels in the direction perpendicular to the panel plane. For both panels E2 and E1, the MWCNTs are well dispersed between the plies. Details for these panels can be seen in the FESEM images of Figures S5 and S6 related to the panels E1 and E3 in the section perpendicular to the plane at magnification higher than those shown in Figure S4. A significant presence of carbon nanotubes arranged through the section perpendicular to the plane of the panel is observed for all the manufactured panels. The panels E1, E2 and E3 were analyzed by TUNA analysis in order to examine the dispersion of carbon nanotubes between the plies of the panels and also to identify the presence of conductive nanodomains through the detection of current values. In particular, CFRC strips were analyzed before and after the etching procedure for a more effective understanding of eventual differences in the morphological characteristics. All CFRC strips were cut out from the panels and analyzed in direction perpendicular to the plane of the panels. Acquisitions using TUNA technique were carried out to evaluate the validity of the implemented infusion process. Figure 12, Figure 13, Figure 14, Figure 15, Figure 16, Figure 17 and Figure 18 and Figures S7–S13 in Supplementary Materials show the strips (both etched and non-etched) of panels E1, E2 and E3 in four modalities of the TUNA images, namely height, deflection error, friction and TUNA current, in 2D and 3D profiles. Figure 12 and Figure S7 show the 2D and 3D profiles of the TUNA images of the etched seven-ply E1 panel strings, respectively. The plies of the panel can be clearly distinguished thanks to the removal of the resin (amorphous phase) determined by the etching procedure. The height images clearly show the fibers of the plies; they are bright yellow if located higher up on the surface and red amaranth if located more in-depth, as deducible from the color bar representative of the height of the different zones under investigation. The carbon nanotubes, discovered on the surface because of the etching procedure, appear almost uniformly dispersed and firmly attached to the carbon fibers, covering almost the entire surface. From the images of the etched samples, it is possible to observe a massive presence of nanotubes in the regions between the fibers of the carbon fabric, where the nanofilled resin is forced by the pressure (see scheme of Figure 1b) to flow through the section of the panel in the direction perpendicular to the plane. This is most likely due to the adopted modified RFI process. The presence of conductive nanoparticles is very evident in the TUNA current micrographs where the carbon nanotubes appear in the form of light-yellow dots that become needles of light-yellow in the corresponding 3D profile, and where low current values ranging from −1.6 to 1.6 pA are detectable, thus confirming the relevant electrical conductivity manifested by the sample. Furthermore, the carbon nanotubes remain adhered to the carbon fibers, despite the treatment of the sample with a strong oxidizing solution. Of course, the etching has been performed only to better analyze the samples, and eventual application of the formulated panels does not require the etching treatment. For this reason, TUNA images were also captured for the non-etched E1 panel to better investigate the map of the electrical conductivity in the as-prepared samples. Figure 13 and Figure S8 show the 2D and 3D profiles, respectively, of the TUNA images of the non-etched strings of E1 panel at the same magnification used in Figure 12 and Figure S7. In this case, the fibers of the carbon fabric are statistically less evident, as the resin, contrary to what was observed in the etched E1 panel, covers them. From the TUNA images, it is possible to observe carbon nanotubes intimately linked to the epoxy matrix and attached to the carbon fibers in such a way as to constitute a single continuous phase that also extends into proximity of the cut layers of carbon fiber located on the left side of each TUNA image. The formation of a conductive network of carbon nanotubes that extends over the entire surface of the sample appears particularly evident in the TUNA current images where it is possible to detect low current values ranging from −1.6 to 1.2 pA. Furthermore, in the TUNA images, the extent and uniformity of the yellow color highlight that the resin impregnating the fibers is electrically conductive in all the observed areas.

Figure 14 and Figure S9 show the 2D and 3D profiles of the TUNA images of the etched strings of panel E2, respectively. Despite the same etching procedure performed on the panel E1, the etched panel E2 (consisting of 14 plies) still contains carbon nanotubes embedded in the resin. The higher number of plies in panel E2 (14 plies) with respect to the panel E1 (7 plies) reduces the efficacy of the etching. This is most likely due to poorer penetration of the oxidizing liquid in the inner central regions of the panel. The TUNA current images show the presence of more light-yellow colored conductive zones corresponding to nanodomains with higher density of carbon nanotubes. The current values measured are in the range between −118.6 and 138.1 pA. Also in this panel, the carbon nanotubes are placed between the carbon fiber plies and intimately linked to the epoxy matrix. In this way, they seem to constitute a single conductive phase, as it can be seen by observing the extensive yellow area between the various bundled layers of carbon fiber which, in the height images, appears deeper and, precisely for this reason, most preserved by the final action of the etching procedure. TUNA images acquired on the non-etched panel E2 further confirm these results. In this regard, Figure 15 and Figure S10 show, respectively, the 2D and 3D profiles of the TUNA images of the non-etched strings of panel E2 at the same magnification used in Figure 14 and Figure S9. The presence of a single conductive phase, consisting of resin incorporating MWCNTs, is easily identifiable in the TUNA current images where it is possible to measure values of low current ranging between −5.4 and 1.7 pA. Figure 16 and Figure S11 show, respectively, the 2D and 3D profiles of the TUNA images of the etched strings of the panel E2 at higher magnification with respect to the previous images. The prevalent presence of conductive nanodomains that cover almost the entire area of the sample is observed, but at this magnification on nanometric scale, it is also possible to observe where the CNTs are exactly located in the resin between the CFs (see the TUNA current image). The regions of the resin, where carbon nanotubes are locally embedded, vary from light-yellow color to light-pink color in the regions with the highest conductivity. The areas with higher conductivity are those appearing more in-depth, in the height images, and therefore the region most preserved by the action of the etching procedure. In any case, the etching action has made it possible to detect an effective dispersion of the conductive nanofiller even in deeper areas strictly connected to the fibers of the carbon fabric. This effect is particularly evident in the 3D TUNA current images where the carbon nanotubes appear of a bright and very clear color (Figure S11). From the TUNA current images of Figure 16 and Figure S11, it can be easily observed that the measured currents fall within the range between −154.5 and 166.6 pA. Figure 17 and Figure S12 show the 2D and 3D profiles of the TUNA images of the etched strings of panel E3, respectively. As expected, since with this sample we have switched to 24 plies of carbon fibers, with the TUNA images it is not possible to observe bare carbon fibers of the carbon fabric. The formation of a continuous conductive network is observable also in the etched panel strips. The TUNA current images highlight a continuous straw-yellow conductive surface associated with extremely low current values between −333.6 and 335.9 fA, thus demonstrating the extreme sensitivity of the TUNA technique in the detecting currents as low as in the order of the femtoampere. Figure 18 and Figure S13 show the 2D and 3D profiles of the TUNA images of the non-etched strings of panel E3, respectively. This nano-filled resin mantle seems more evident in the TUNA current images. Here, the carbon nanotubes appear in the form of white cords that extend along, and appear to twist around, the layers of carbon fibers. As observed for the etched panel E3, even for the non-etched panel E3, very low values of current ranging from −871.3 to 409.6 fA have been measured.

### 3.6. Electrical Properties

#### Effect of the Plies Number on the Electrical Properties

The DC characterization of all analyzed samples shows an ohmic behavior. The linear dependence of the measured voltage (*V*_2_) with respect to the applied current (*I*_H_ for strip and *I*_t_ for square sample, ranging from 0–1 A with current step of 50 mA up to 500 mA and step of 100 mA up to 1 A) is used to derive the resistance of the sample, i.e., *V*_2_ = *R*_c_ × *I*. Table 4 summarizes the results achieved from the setup measurements of Figure 2 applied on three CFRC strip samples obtained by cutting samples aligned to the fiber′s direction from E1 panel, useful to derive the equivalent parallel (σ_//0°_) in-plane conductivity. The conductivity is derived from Equation (2) with uncertainty (Δσ) due to the measurement chain whose value also reported in Table 4. Here, the very high R^2^ obtained by considering a confidence interval of 95% of the interpolation line is also stated. Table 5 summarizes the detected DC conductivities for the panels E1, E2 and E3, obtained by measuring five samples for each configuration and applying Equation (2) (for σ_//0°_ and σ_//45°_), Equation (6) (for σ_﬩_) and Equation (10) (for σ_s_).

**Figure 19:**
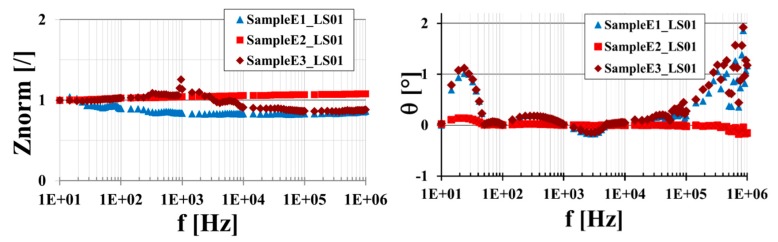
The module of the equivalent impedance normalized to Z_@10Hz_ (**left**) and phase (**right**) for the large square samples with different number of plies.

By increasing the number of plies, the in-plane conductivity σ_//45°_ is quite constant for systems with 7 and 14 plies (E1 and E2), whereas it increases by 18% for that with 24 layers (E3). Also, the out-of-plane conductivity σ_﬩_ does not change significantly for E1 and E2, whereas it shows a very large (over thirty times) increment for the panel E3. For the interpretation of this different behavior it should be considered that the out-of-plane conductivity depends on the conduction between different plies. The impregnation of the carbon fibers, whose conductivity is some tens of kS/m with a nanofilled resin characterized by a lower conductivity of hundreds of mS/m, induces a decrease of the conduction between the plies. For the system with 24 plies, a lower amount of nanofilled matrix between the (inner) CF plies may have a less pronounced impact on the conduction mechanism, therefore leading to the large value of the “equivalent” conductivity detected for system E3. Due to the same mechanism (i.e., the increasing impact of the conductivity of the impregnating resin with the number of CF layers) the increase in the plies number corresponds to a decrease in the in-plane σ_//0 °_. Such a reduction is 22% by moving from 7 to 14 plies and is 12% from 14 to 24. Moreover, the increase in the plies number is effective on the surface conductivity: E2 and E3 systems exhibit a more than 16 times lower surface conductivity than that of E1. Once more this effect can be justified by considering that in E2 and E3 preparation, due to the more difficult impregnation process, the nanofilled resin tends to accumulate on the surface. By summarizing the DC electrical characteristics reported in Table 5, the highest equivalent bulk conductivity is shown by the E1 panel. In particular, the E1 system exhibits a 16.19 ± 2.6% (kS/m) of in-plane conductivity in the parallel direction to the carbon fibers. In the case of fiber cutting at 45°, the 24 plies (E3) demonstrates a conductivity of 14.26 ± 2.1% (kS/m), which is about 15% higher than that of the E1 system. However, if the values at 0° and 45° are averaged, the value obtained for the E1 system is 14.1 kS/m, which is about 13% higher than that equal to 12.5 (kS/m) exhibited by the E3 system. Furthermore, E1 and E2 systems exhibit the lowest out-of-plane conductivity. In order to compare the AC behavior of the different systems, the frequency analysis has been performed in the range of 10 Hz to 1 MHz. Normalization of the measured impedance with respect to the value at 10 Hz, i.e., *Z*_norm_ = *Z*/*Z*_@10Hz_, is considered. Figure 19 illustrates the comparison of the module and phase of the normalized impedance for the three systems (E1, E2 and E3) obtained by varying the number of plies.

From the data reported in Figure 19, it is possible to observe that the amplitude of the normalized impedance and the phase impedance range in narrow intervals around one (i.e., 0.83–1.04 for E1, 1–1.08 for E2 and 0.86–1.26 for E3 panel) and zero degrees (maximum is 2° at the highest explored frequency for panel E3), respectively. This characteristic, observed independently from the number of plies, corresponds to the very high conductivity exhibited by all systems that, in the considered frequency range, behave as resistive materials.

### 3.7. Effect of GPOSS on the Electrical Properties

The mixture of GPOSS in the matrix provides a multifunctional CFRCs with enhanced flame-retardant properties. In order to investigate the influence of the GPOSS filler (at 5 wt % loading) on the electrical behavior of the CFRCs, the performance of the E1 system (which includes the GPOSS) has been compared with a similar system (named D) with seven plies impregnated with the same MWCNT-epoxy resin in which the GPOSS is not present. The measured values of the in-plane and out-of-plane conductivities used to ascertain the impact of the GPOSS are summarized in Table 6.

**Figure 20:**
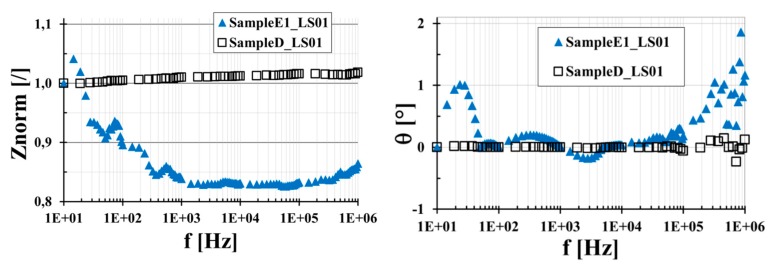
The module of the equivalent impedance normalized to Z_@10Hz_ (**left**) and phase (**right**) for seven-ply CFRCs obtained with (sampleE1_LS01) and without (sampleD_LS01) 5 wt % of GPOSS flame retardant filler.

The system without GPOSS (sample D) shows higher bulk electrical conductivities than those related to the E1 system. In particular, the inclusion of GPOSS leads to a reduction of about 13% of the in-plane and about 35% of the out-of-plane conductivity. On the other side, GPOSS has a positive influence on the surface conductivity since system E1 shows a 5-times increase with respect to that measured for the system D. This result is most likely due to the lower viscosity of the formulation containing GPOSS, which allows a better impregnation process. Due to the very high values of the conductivities, the frequency behavior of the equivalent materials is governed by the resistive part, with Znorm ranging around 1 and phase almost equal to 0°. In Figure 20, the normalized impedance of the E1 and D systems is reported showing that, in this sense, the GPOSS does not affect the frequency response of the sample; it still remains as that of a resistor.

## 4. Conclusions

In this paper, new CFRCs are proposed with the aim to have a load-bearing structure with functional properties for lightning protection. In particular, MWCNTs improve the electrical conductivity of the manufactured laminates and GPOSS acts as a flame retardant. The highest value of DC conductivity (σ_//0_ and σ_﬩_) was measured for system D (seven plies, without GPOSS, with 0.5% of MWCNTs and “bulk infusion”). The AC behavior of this formulation is characterized by high electrical stability in the explored frequency range, showing a resistive response of the system in the out-of-plane direction, where the effect of the impregnating resin is dominant. This is a very promising result strictly related to the choice of the manufacturing methodology described in the paper. The solubilization of GPOSS in the matrix (systems E1, E2 and E3 corresponding to 5 wt % of GPOSS and 0.5 wt % of MWCNTs with 7, 14 and 24 plies, respectively) provides multifunctional panels characterized by relevant functions integrated into the same panel (high electrical conductivity and flame resistance). Electrical tests performed on this last panel (E series) also evidence that the presence of the flame retardant (GPOSS) preserves its AC electrical stability. Besides, for the first time, CFRC strings were analyzed by tunneling atomic force microscopy (TUNA) technique which allowed the identification of the nanometric arrangement of the conductive phase and features of the charge conduction through the current pathways in the CNT-based panels. In this work, the conductive paths are represented by carbon nanotubes that are strongly attached to the carbon fibers with the peculiar tendency to accumulate in the areas through which the passage of the resin takes place due to the particular infusion process. For all the manufactured panels, TUNA current images point out the presence, between the layers of carbon fibers, of conductive networks of carbon nanotubes that contribute effectively to guarantee the good electrical performance of the manufactured panels.

## Figures and Tables

**Table 1:** Epoxy matrix formulations.

Sample	CNTs (wt %)	GPOSS (wt %)
Pristine	-	-
Formulation D	0.5	-
Formulation E	0.5	5.0

**Table 2:** Characteristics and composition of the materials manufactured using the bulk infusion process.

Sample	No. of Plies	Resin Composition
Sample D	7	T25BD + 0.5% MWCNT
Sample E1	7	T25BD + 5% GPOSS + 0.5% MWCNT
Sample E2	14	T25BD + 5% GPOSS + 0.5% MWCNT
Sample E3	24	T25BD + 5% GPOSS + 0.5% MWCNT

**Table 3:** Curing degree of the formulation E and the manufactured panels.

Sample	Curing Degree (%)
Formulation E (from the first run)	100
E1 panel	94
E2 panel	96
E3 panel	96

**Table 4:** Summary of the results obtained from the measurements on three E1 strip samples.

Samples E1	*R*_c_ (mΩ)	σ_//0°_ (S/m)	Δσ_//0°_ (S/m)	Δσ_//0°_ (%)	R^2^
ST00_05	53.73	1.608 × 10^4^	4.1 × 10^2^	2.57	0.99
ST00_06	49.82	1.545 × 10^4^	4.0 × 10^2^	2.61	0.99
ST00_07s	47.39	1.704 × 10^4^	4.5 × 10^2^	2.66	0.99

**Table 5:** Bulk and surface conductivities for systems E1, E2 and E3.

Sample	E1	E2	E3
No. of plies	7	14	24
σ_//0°_ (kS/m)	16.19	12.29	10.82
Δσ_//0°_ (%)	2.6	2.3	2.2
σ_//45°_ (kS/m)	12.10	12.06	14.26
Δσ_//45°_ (%)	2.4	2.3	2.1
σ_﬩_ (S/m)	0.06	0.11	1.75
Δσ_﬩_ (%)	4.0	3.5	2.5
σ_s_ (mS)	55.50	3.35	3.31
Δσ_s_ (%)	4.7	4.7	5.5

**Table 6:** Maximum in-plane and out-of-plane conductivities for the seven-ply CFRCs obtained without (D) and with (E1) 5 wt % of GPOSS flame retardant filler.

Sample	No. of Plies	σ_//0_ (S/m)	σ_﬩_ (S/m)	σ_s_ (S/m)
Sample D	7	1.950 × 10^4^	3.85	0.011
Sample E1	7	1.703 × 10^4^	0.11	0.055

## Supplementary Materials

The following are available online at https://www.mdpi.com/2073-4360/11/11/1865/s1, Figure S1: (a) Resin thick film distribution; (b) Preform on the thick resin film; (c) Breather/bleeder around the preform; (d) Laminated panels in autoclave; (e) Squeezing of resin and final result; (f) Final Panel (E2); Figure S2: FESEM images of the nanocomposite TBD+5%GPOSS+0.5%MWCNT (formulation E); Figure S3: EDX images of the nanocomposite TBD+5%GPOSS+0.5%MWCNT (formulation E); Figure S4: FESEM images of the panel E2 (on the left side) and panel E1 (on the right side); Figure S5: FESEM images of the panel E1; Figure S6: FESEM images of the panel E3; Figure S7: TUNA micrographs (Height, Deflection Error, Friction, TUNA Current profiles in 3D) of the etched panel E1; Figure S8: TUNA micrographs (Height, Deflection Error, Friction, TUNA Current profiles in 3D) of the non-etched panel E1; Figure S9: TUNA micrographs (Height, Deflection Error, Friction, TUNA Current profiles in 3D) of the etched panel E2; Figure S10: TUNA micrographs (Height, Deflection Error, Friction, TUNA Current profiles in 3D) of the non-etched panel E2; Figure S11: TUNA micrographs at higher magnification (Height, Deflection Error, Friction, TUNA Current profiles in 3D) of the etched panel E2; Figure S12: TUNA micrographs (Height, Deflection Error, Friction, TUNA Current profiles in 3D) of the etched panel E3; Figure S13: TUNA micrographs (Height, Deflection Error, Friction, TUNA Current profiles in 3D) of the non-etched panel E3.
