# Supplementary material for: Electrical Current Map and Bulk Conductivity of Carbon Fiber-Reinforced Nanocomposites
Source: Polymers (Basel). 2019 Nov 12;11(11):1865. doi: 10.3390/polym11111865 (PMC6918280; doi:10.3390/polym11111865)
Supplement: Supplementary file 1 [file polymers-11-01865-s001.pdf]

Supplementary electronic materials

Electrical current map and bulk conductivity of carbon fiber reinforced nanocomposites

Liberata Guadagno[1]*, Luigi Vertuccio[1], Carlo Naddeo[1], Marialuigia Raimondo[1]*, Giuseppina Barra[1], Felice De Nicola[2], Ruggero Volponi[2], Patrizia Lamberti[3], Giovanni Spinelli[3], Vincenzo Tucci[3]

1 Department of Industrial Engineering, University of Salerno, Via Giovanni Paolo II, 132, 84084, Fisciano (SA), Italy; lguadagno@unisa.it (L.G.); lvertuccio@unisa.it (L.V.); cnaddeo@unisa.it (C.N.); mraimondo@unisa.it (M.R.); gbarra@unisa.it (G.B.)

2 CIRA Italian Aerospace Research Centre, Advanced Materials and Technologies Lab, Via Maiorise, Capua (CE), Italy; fdenicola@cira.it (F.D.); rvolponi@cira.it (R.V.)

3 Department of Information and Electrical Engineering and Applied Mathematics, University of Salerno, Via Giovanni Paolo II, 132, 84084, Fisciano (SA), Italy; plamberti@unisa.it (P.L.); gspinelli@unisa.it (G.S.); vtucci@unisa.it (V.T.)

* Correspondence: lguadagno@unisa.it (L.G.); Tel: +39 089 964114; mraimondo@unisa.it (M.R.); Tel: +39 089 964019

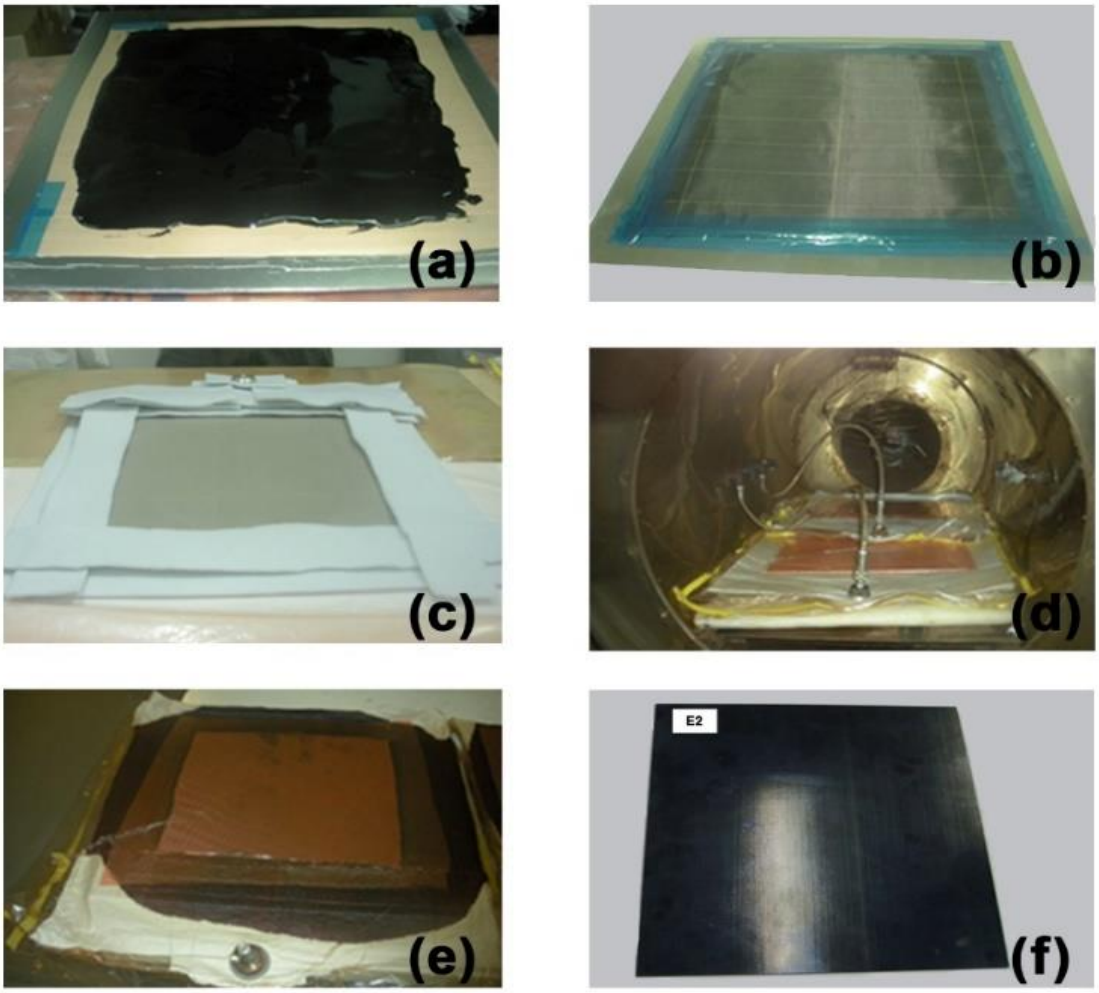

Figure S1. a) Resin thick film distribution; b) Preform on the thick resin film; c) Breather/bleeder around the preform; d) Laminated panels in autoclave; e) Squeezing of resin and final result; f) Final Panel (E2).

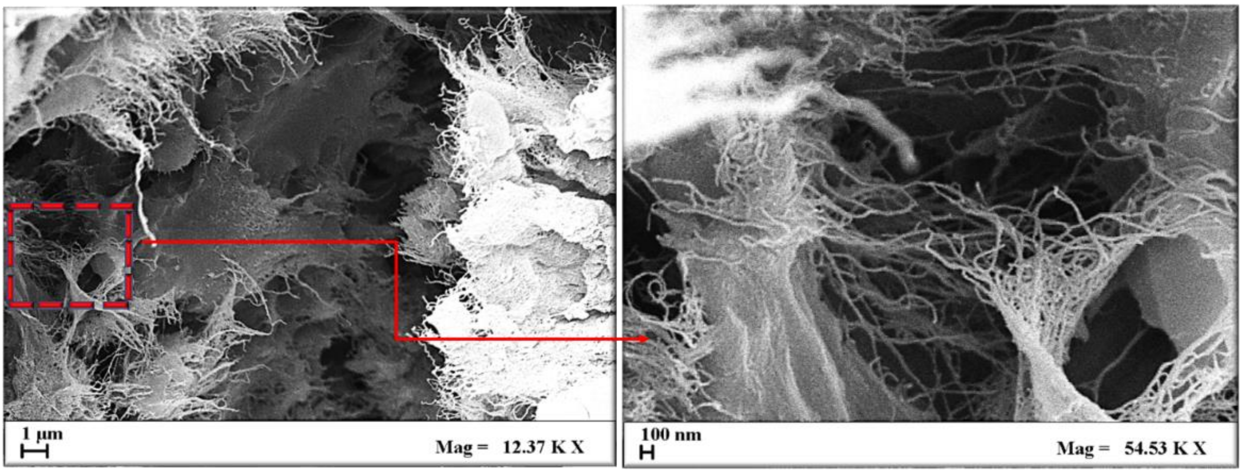

Figure S2. FESEM images of the nanocomposite TBD+5%GPOSS+0.5%MWCNT (formulation E).

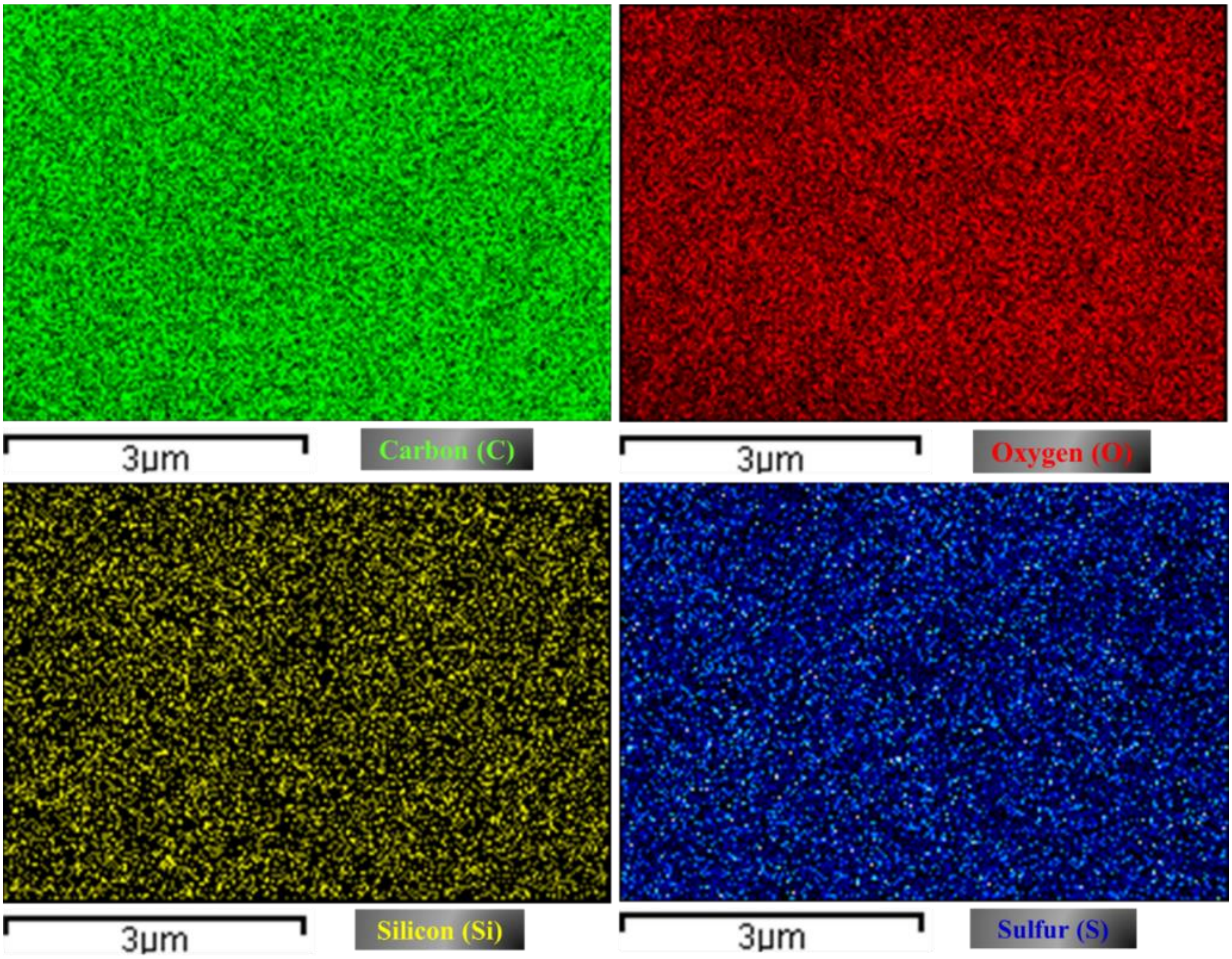

Figure S3. EDX images of the nanocomposite TBD+5%GPOSS+0.5%MWCNT (formulation E).

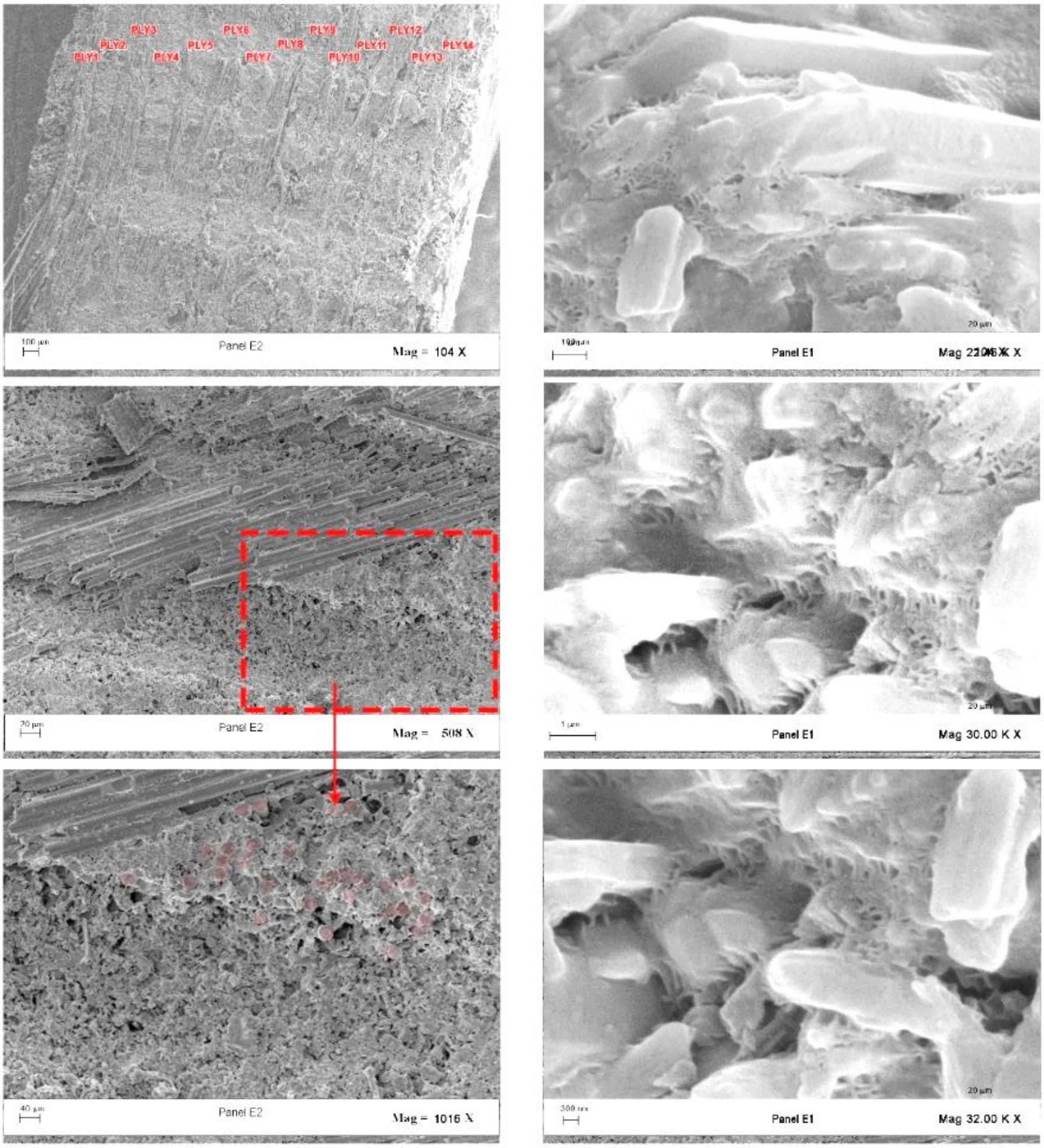

Figure S4. FESEM images of the panel E2 (on the left side) and panel E1 (on the right side).

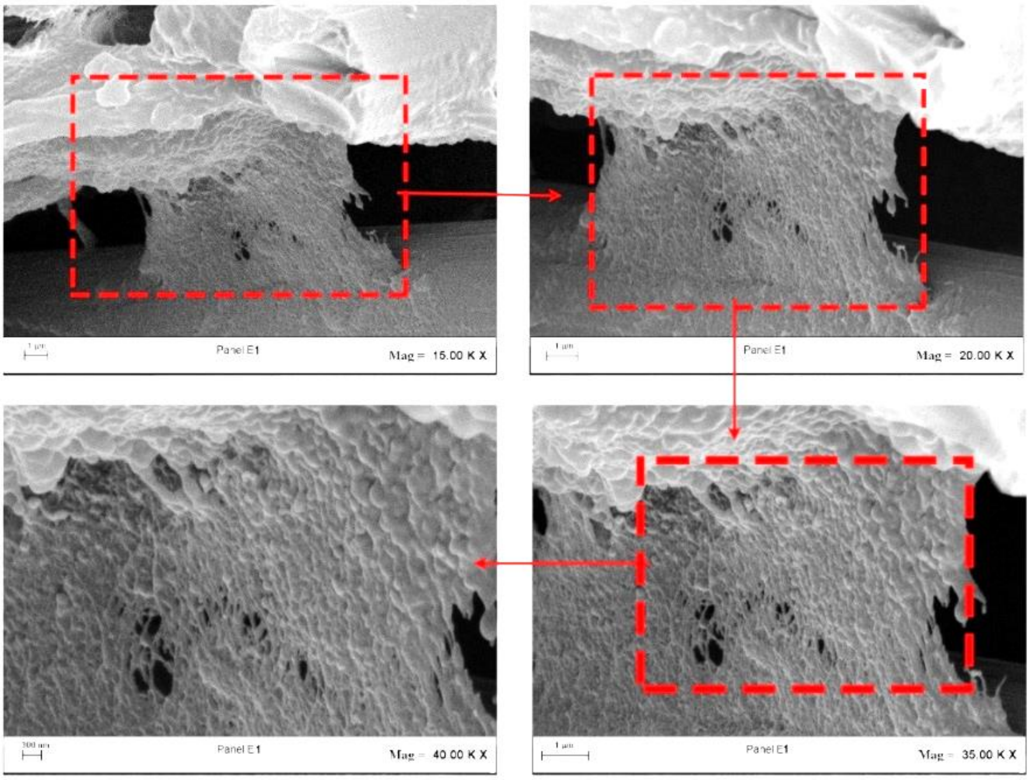

Figure S5. FESEM images of the panel E1.

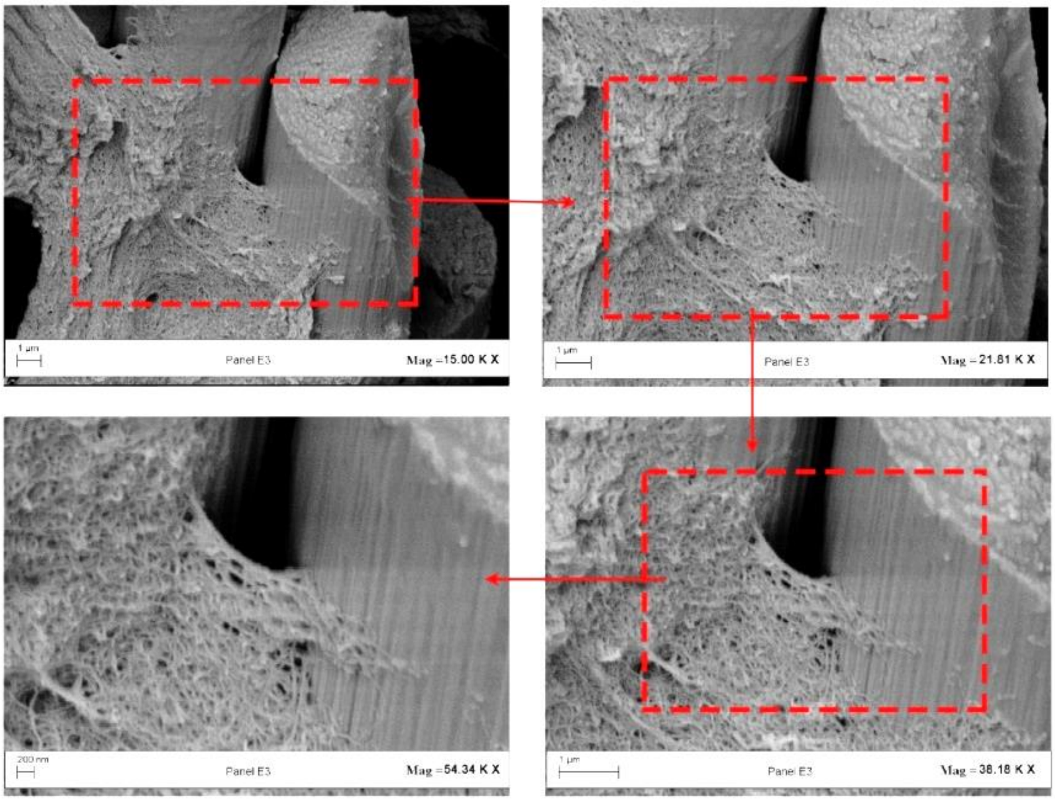

Figure S6. FESEM images of the panel E3.

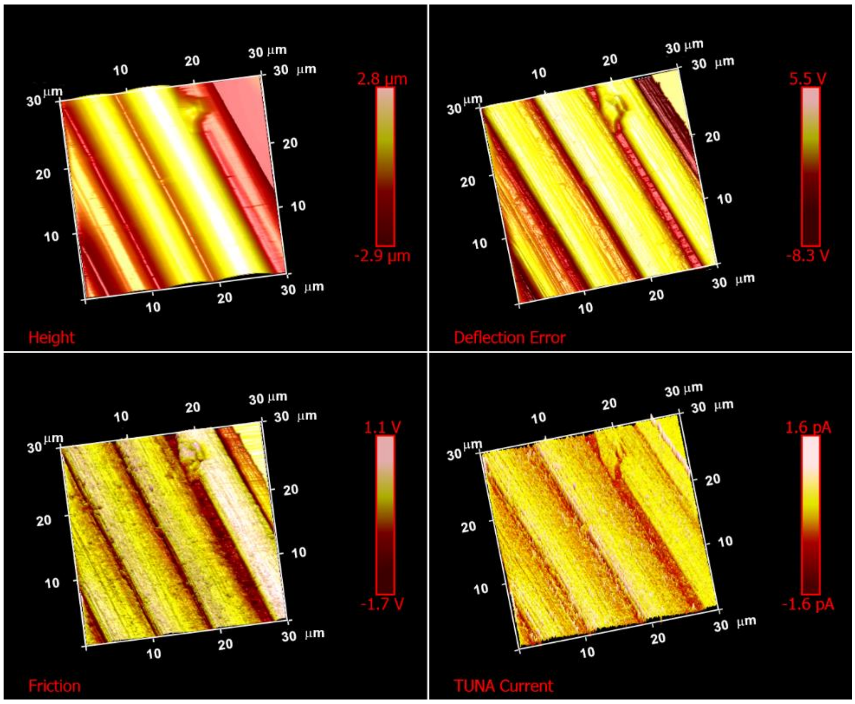

Figure S7. TUNA micrographs (Height, Deflection Error, Friction, TUNA Current profiles in 3D) of the etched panel E1.

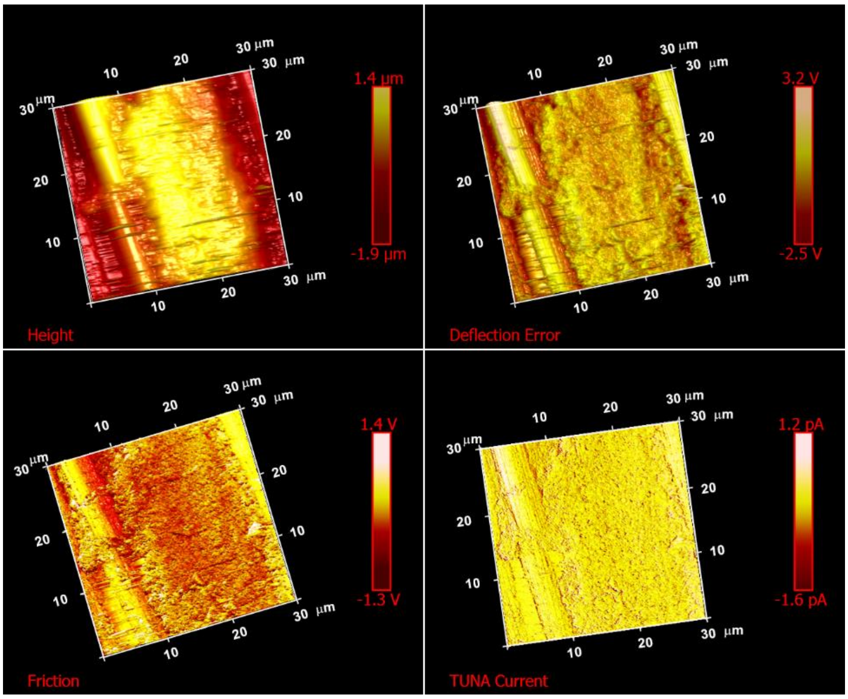

Figure S8. TUNA micrographs (Height, Deflection Error, Friction, TUNA Current profiles in 3D) of the non-etched panel E1.

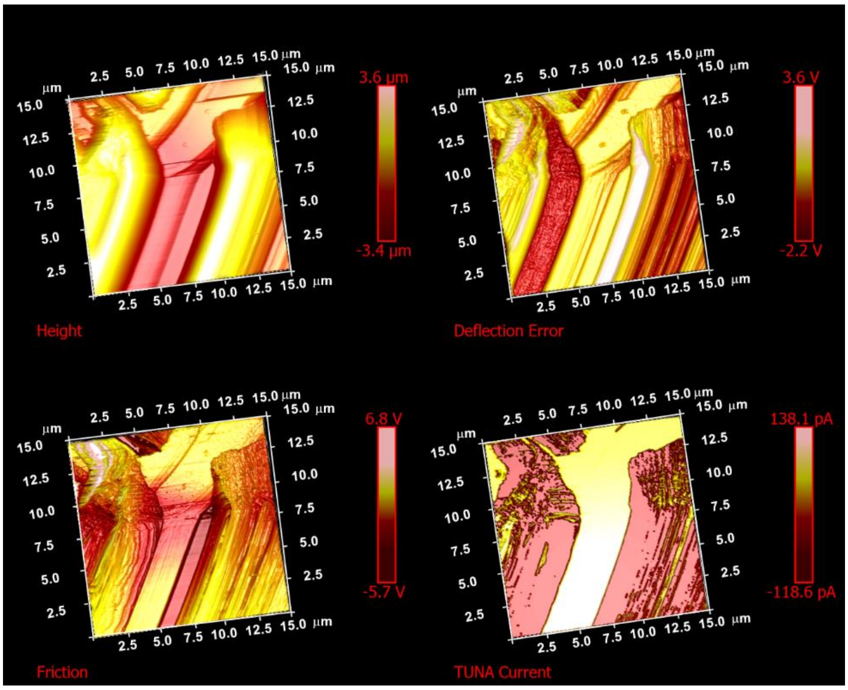

Figure S9. TUNA micrographs (Height, Deflection Error, Friction, TUNA Current profiles in 3D) of the etched panel E2.

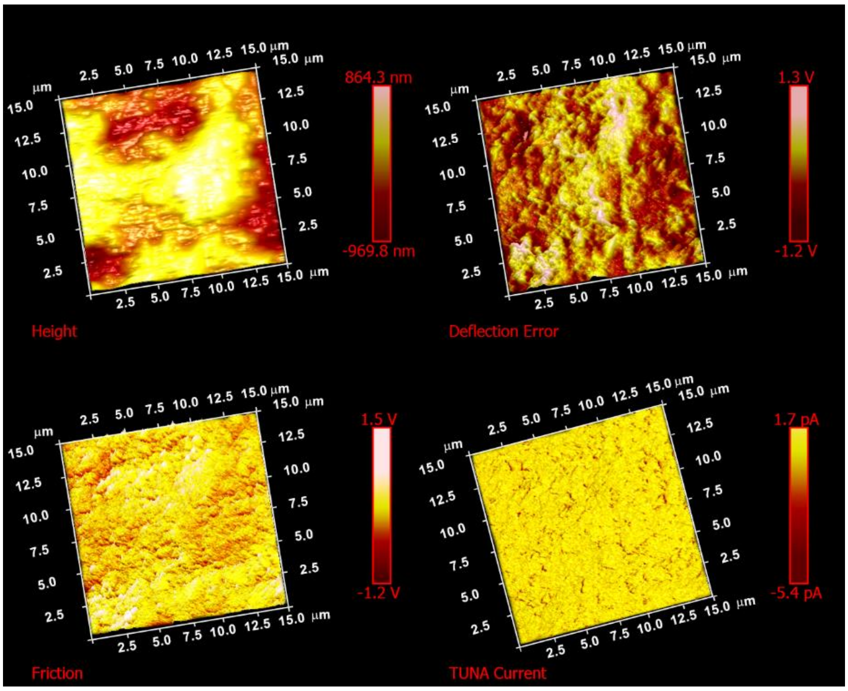

Figure S10. TUNA micrographs (Height, Deflection Error, Friction, TUNA Current profiles in 3D) of the non-etched panel E2.

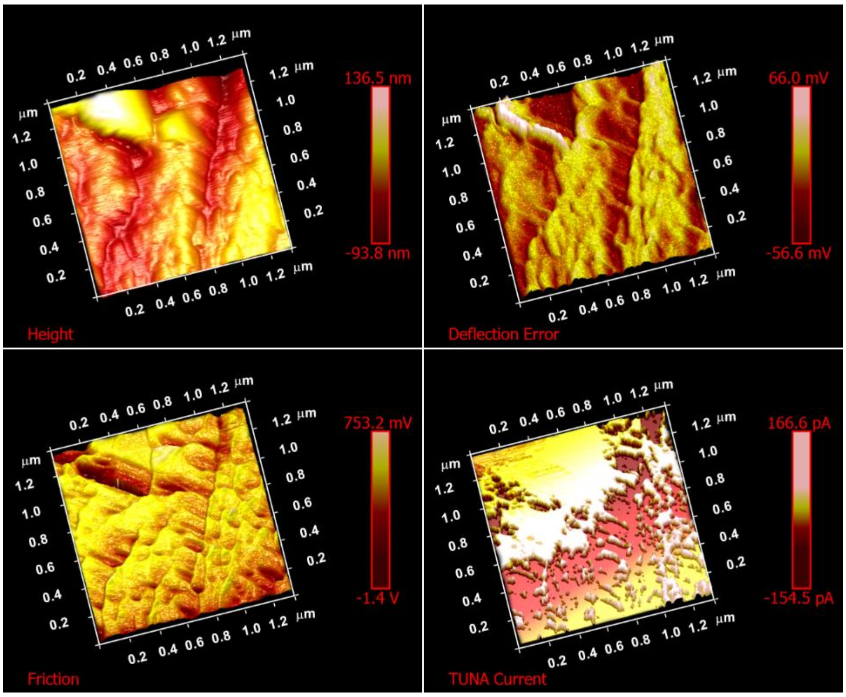

Figure S11. TUNA micrographs at higher magnification (Height, Deflection Error, Friction, TUNA Current profiles in 3D) of the etched panel E2.

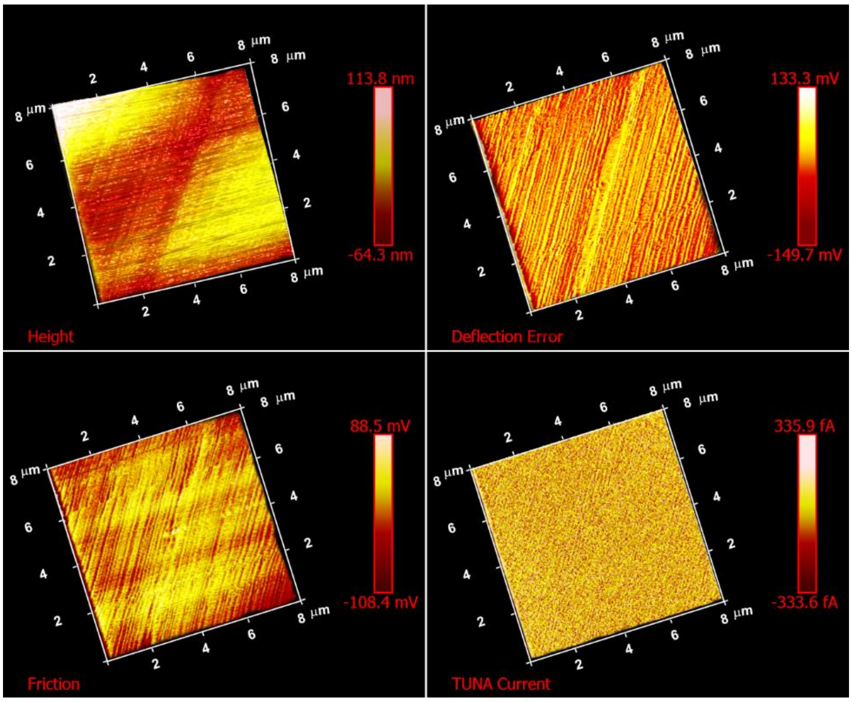

Figure S12. TUNA micrographs (Height, Deflection Error, Friction, TUNA Current profiles in 3D) of the etched panel E3.

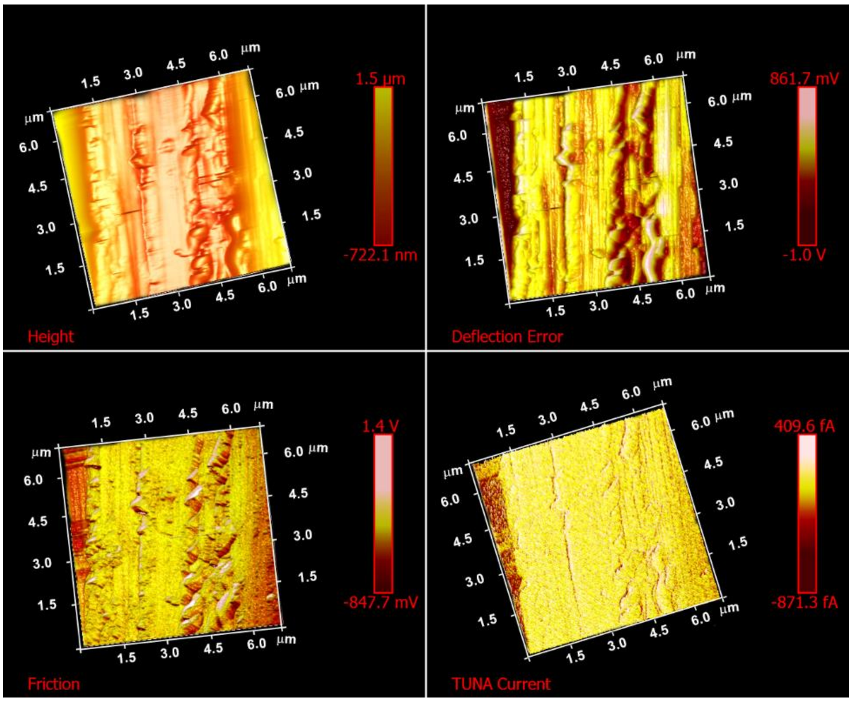

Figure S13. TUNA micrographs (Height, Deflection Error, Friction, TUNA Current profiles in 3D) of the non-etched panel E3.
